# Immuno-genomic classification of colorectal cancer organoids reveals cancer cells with intrinsic immunogenic properties associated with patient survival

**DOI:** 10.1186/s13046-021-02034-1

**Published:** 2021-07-13

**Authors:** Eun Jeong Cho, Minsuh Kim, Daum Jo, Jihye Kim, Ji-Hye Oh, Hee Chul Chung, Sun-hye Lee, Deokhoon Kim, Sung-Min Chun, Jihun Kim, Hyeonjin Lee, Tae Won Kim, Chang Sik Yu, Chang Ohk Sung, Se Jin Jang

**Affiliations:** 1 Asan Center for Cancer Genome Discovery, Asan Institute for Life Sciences, Asan Medical Center, Seoul, Republic of Korea; 2 Department of Medical Science, Asan Medical Institute of Convergence Science and Technology, Asan Medical Center, University of Ulsan College of Medicine, Seoul, Republic of Korea; 3 Asan Institute for Life Sciences, Asan Medical Center, Seoul, Republic of Korea; 4 Department of Pathology, Asan Medical Center, University of Ulsan College of Medicine, 88 Olympic-ro 43-gil, Songpa-gu, Seoul, 05505 Republic of Korea; 5 Department of Oncology, Asan Medical Center, University of Ulsan College of Medicine, Seoul, Republic of Korea; 6 Department of Colon and Rectal Surgery, Asan Medical Center, University of Ulsan College of Medicine, Seoul, Republic of Korea; 7 OncoClew Life Science Co., Ltd, Songpa-gu, Seoul, Republic of Korea

**Keywords:** Colorectal cancer, Organoids, Gene expression, Intrinsic, HLA-II, Immuno-genomic, Microenvironment, Prognosis

## Abstract

**Background:**

The intrinsic immuno-ge7nomic characteristics of colorectal cancer cells that affect tumor biology and shape the tumor immune microenvironment (TIM) are unclear.

**Methods:**

We developed a patient-derived colorectal cancer organoid (CCO) model and performed pairwise analysis of 87 CCOs and their matched primary tumors. The TIM type of the primary tumor was classified as immuno-active, immuno-exhausted, or immuno-desert.

**Results:**

The gene expression profiles, signaling pathways, major oncogenic mutations, and histology of the CCOs recapitulated those of the primary tumors, but not the TIM of primary tumors. Two distinct intrinsic molecular subgroups of highly proliferative and mesenchymal phenotypes with clinical significance were identified in CCOs with various cancer signaling pathways. CCOs showed variable expression of cancer-specific immune-related genes such as those encoding HLA-I and HLA-II, and molecules involved in immune checkpoint activation/inhibition. Among these genes, the expression of HLA-II in CCOs was associated with favorable patient survival. K-means clustering analysis based on HLA-II expression in CCOs revealed a subgroup of patients, in whom cancer cells exhibited Intrinsically Immunogenic Properties (Ca-IIP), and were characterized by high expression of signatures associated with HLA-I, HLA-II, antigen presentation, and immune stimulation. Patients with the Ca-IIP phenotype had an excellent prognosis, irrespective of age, disease stage, intrinsic molecular type, or TIM status. Ca-IIP was negatively correlated with intrinsic E2F/MYC signaling. Analysis of the correlation between CCO immuno-genotype and TIM phenotype revealed that the TIM phenotype was associated with microsatellite instability, Wnt/β-catenin signaling, *APC/KRAS* mutations, and the unfolded protein response pathway linked to the *FBXW7* mutation in cancer cells. However, Ca-IIP was not associated with the TIM phenotype.

**Conclusions:**

We identified a Ca-IIP phenotype from a large set of CCOs. Our findings may provide an unprecedented opportunity to develop new strategies for optimal patient stratification in this era of immunotherapy.

**Supplementary Information:**

The online version contains supplementary material available at 10.1186/s13046-021-02034-1.

## Background

Understanding the intrinsic immuno-genomic characteristics of cancer cells is essential if we are to develop optimal patient stratification systems for targeted therapy. Moreover, the tumor immune microenvironment (TIM) has received significant attention due to its close association with responses to immunotherapy. However, baseline data are lacking, and few studies have examined the intrinsic immuno-genomic properties of patient-derived cancer cells. Currently, comprehensive characterization of cancers is based on gene expression patterns in bulk cancer tissues, which contain both cancer cells and the TIM. RNA sequencing delineates the global gene expression pattern in primary cancer tissues as it captures cancer cells as well as various cells in the TIM; therefore, gene expression patterns in cancer tissues reflect both these entities [1–3]. However, the TIM is “noisy” and can mask intrinsic cancer-specific signatures in bulk primary tissues. Therefore, an analysis based on the detection of purely intrinsic cancer cell-derived signals will highlight the immuno-genomic characteristics of the cancer cells themselves. To acquire such purely intrinsic signals, it is necessary to use well-defined cancer models rather than cancer cell lines, as the former will recapitulate cancer tissues in the absence of the TIM.

Recently, tissue-specific stem cells from several human organs were cultured to generate organoids, which mimic the 3D structure and function of the organ from which they are derived [4–7]. These organoid models have been developed for several cancers and used successfully to recapitulate the architecture of primary cancer tissues [8–11]. Organoids derived from colorectal cancer have been shown to maintain the therapeutic responses as well as various genomic characteristics of the primary tumor [12–15]. However, these studies have included only a small number of organoids, and studies investigating the collective features of colorectal cancer in large numbers of colorectal cancer organoids (CCOs) are still lacking. Pairwise integrative analysis of CCOs and the corresponding cancer tissues with TIM in a large number of samples can provide deeper insights into the purely intrinsic immuno-genomic characteristics of colorectal cancers.

Here, we generated 87 CCOs as part of an organoid biobank project. These CCOs closely mimicked the histological characteristics of their respective primary cancers. We then evaluated the cancer-specific intrinsic immuno-genomic properties of colon cancer cells using this large set of CCOs and their corresponding cancer tissues after confirming that these organoids recapitulated the signatures of the primary cancers at the transcriptome level.

## Materials and methods

### Patient samples

Small (1–4 cm^3^) sections of colorectal cancer tissue samples were obtained from resected colorectal specimens as part of the biobanking process for colorectal cancer at the Asan Bio-Resource Center of Asan Medical Center (Seoul, Korea). All tissues were used with the patients’ consent. The research protocol was approved by the Ethics Committee of Asan Medical Center, and the entire experimental protocol was conducted in compliance with the relevant institutional guidelines. Samples were categorized as tumor or normal tissue based on histopathological assessment. The diagnosis of each patient was confirmed by pathologists at Asan Medical Center. A total of 87 colorectal cancers were used to generate the CCO models used in this study. The clinicopathological features of the 87 patients are summarized in **Supplementary Table** **1**.

### Culturing patient-derived cancer organoids

Within 1 h of excision, patient samples were placed in cold Hank’s balanced salt solution (HBSS; Lonza, Basel, Switzerland) containing 1× primocin (Invivogen, Hong Kong, China) and transported to the laboratory on ice. Samples were then washed three times with cold HBSS containing antibiotics and cut into 1–2 mm^3^ sections using sterile blades. The sectioned samples were incubated (37 °C for 40–90 min with intermittent agitation) in DMEM/F12 medium (Gibco, OK, USA) supplemented with 0.2 U/μL collagenase II (Gibco), 1% penicillin/streptomycin (Gibco), and 0.5 mg/mL amphotericin B (2%; Sigma-Aldrich, MO, USA). After incubation, the suspensions were triturated repeatedly by pipetting, centrifuged at 1200 rpm for 5 min, and washed three times with DPBS (Welgene). Next, the suspensions were passed through 100 μm cell strainers (BD Falcon, CA, USA) and centrifuged at 600 rpm for 3 min. The resulting pellet was resuspended in 100 μL minimum basal medium for CCO (serum-free medium [DMEM/F12; Gibco] supplemented with 50 ng/mL human epidermal growth factor [Invitrogen, CA, USA], B27 [Invitrogen], 1 mM N-acetylcysteine [Peprotech, NJ, USA], 10 mM nicotinamide [Peprotech], 10 nM gastrin I [Peprotech], 500 nM A83–01 [Peprotech], 10 μM ROCK inhibitor [Peprotech], and 1% penicillin/streptomycin [Gibco]). Then, 200 μL Matrigel (Corning, NY, USA) was added to the remaining 100 μL suspension to establish organoids, and 150 μL of the resulting cell suspension was allowed to solidify in a single well of a 6-well culture plate (Corning) pre-warmed at 37 °C for 10 min. After gelation, 3 mL CCO MBM was added to the well, and the medium was changed every 4 days. The organoids were passaged after 1–3 weeks. For passage, a solidified Matrigel drop containing the organoids was harvested in cold DPBS and then centrifuged at 112×*g* for 3 min at 4 °C. The pellets were washed with cold DPBS and centrifuged at 250 rcf for 15 min at 4 °C, and the organoids in the pellet were resuspended in 2 mL TrypLE Express (Invitrogen) and incubated for 10 min at 37 °C to allow dissociation. After this, 10 mL DMEM/F12 containing 10% FBS was added and the mixture centrifuged at 112×g for 3 min. The pellets were then washed with DPBS, centrifuged at 112 g for 3 min, resuspended in CCO MBM + Matrigel (1:3), and reseeded at a ratio of 1:3 or 1:4 to form new CCOs.

### Histology and imaging

Tissues and CCOs (> passage 3) were fixed in 4% paraformaldehyde, followed by dehydration, paraffin embedding, sectioning, and standard haematoxylin and eosin (H&E) staining. For immunohistochemistry (IHC), samples were incubated with primary antibodies specific for carcinoembryonic antigen (CEA; 1:200 dilution; Dako, CA, USA), CDX2 (1:200 dilution; Novocastra, IL, USA), and cytokeratin20 (CK20; 1:400 dilution; Dako). The sections were subsequently incubated with secondary antibodies (#AI-2000 and #AI-1000; 1:5000; Vector Laboratories, CA, USA) and visualized using the ultraView Universal DAB Detection kit (Ventana Medical Systems, AZ, USA). Nuclei were counterstained with Harris haematoxylin. Images were acquired under a Leica Eclipse E600 microscope.

### Tissue microarray construction and immunohistochemical analysis

A tissue microarray was constructed for the primary tumor tissues. Two random cores (2 mm in size) were obtained from formalin-fixed and paraffin-embedded (FFPE) primary tumor tissues after microscopic evaluation. The tissue microarray was subjected to immunohistochemical analysis using antibodies specific for **HLA-DR/DP/DQ/DX (1:1000, mouse monoclonal, clone CR3/43; catalogue No. SC-53302; SantaCruz Biotechnology, CA, USA),** KI-67 (1:200, Mouse monoclonal, clone MIB1; catalogue No. M7240; Dako, Glostrup, Denmark), E2F1 (1:100, Mouse monoclonal, clone KH95; catalogue No. 32–1400; Invitrogen), and cyclin-E (1:400, Mouse monoclonal, clone H212; catalogue No. MA5–14336; Thermo, CA, USA). Briefly, FFPE tissue sections were immunohistochemically stained using an OptiView DAB IHC (immunohistochemistry) Detection Kit and a BenchMark XT automatic immunostaining device (Ventana Medical Systems, AZ, USA). Antigen-antibody reactions were visualized using the Ventana OptiView DAB IHC Detection Kit (OptiView HQ Linker 8 min, OptiView HRP Multimer 8 min, OptiView H_2_O_2_/DAB 8 min, and OptiView Copper 4 min). Counterstaining was performed for 12 min using Ventana Haematoxylin II and for a further 4 min using Ventana Bluing reagent. Finally, all slides were removed from the stainer, dehydrated, and covered with a cover slip prior to microscopic examination.

### Targeted DNA sequencing and data processing

To obtain CCOs (> passage 3) for genomic analysis, a solidified Matrigel drop containing the CCOs was harvested in cold DPBS and centrifuged at 112×*g* for 3 min at 4 °C. The pellets were washed with cold DPBS and centrifuged at 250×*g* for 15 min at 4 °C. Then, genomic DNA was extracted using the DNeasy Blood & Tissue Kit (Qiagen, Germany). A DNA library was prepared using the SureSelect XT custom kit (Agilent Technology) after checking DNA quality. Pooled libraries were sequenced at the Department of Pathology, Asan Medical Center on an Illumina MiSeq instrument (Illumina, CA, USA) using a targeted gene panel. This next-generation sequencing (NGS) system has been approved for clinical NGS testing by the Korean government. The targeted gene panel is approximately 1 Mb in size and contains 33,524 probes targeting 382 genes, including the entire exons of 199 genes, 184 hotspots, and partial introns of eight genes frequently rearranged in cancers [16, 17]. Targeted sequencing of CCOs was performed without matched normal tissue.

Sequenced reads were aligned against the human reference genome (National Center for Biotechnology Information build 37) using BWA (0.5.9) with default options [18]. Demultiplexing was performed using the MarkDuplicates tool in the Picard package (Broad Institute, Cambridge, MA; *http://broadinstitute.github.io/picard*, last accessed on February 14, 2018) to remove PCR duplicates. The deduplicated reads were realigned at known indel positions using the GATK IndelRealigner tool [19]. Next, the base qualities were recalibrated using the GATK BaseRecalibrator tool. Somatic single-nucleotide variants and short indels were detected with an unmatched normal using Mutect version 1.1.6 and the SomaticIndelocator tool in GATK (Broad Institute) [19–21]. Common and germline variants from somatic variant candidates were filtered out using the common dbSNP build 141 (found in > 1% of samples), Exome Aggregation Consortium release 0.3.1 (https://gnomad.broadinstitute.org/), the Korean Reference Genome database (http://coda.nih.go.kr/coda/KRGDB/index.jsp), and an in-house panel of normal variants. Final somatic variants were annotated using Variant Effect Predictor version 79 [22] and converted to the maf file format using vcf2maf (GitHub; *https://github.com/mskcc/vcf2maf*, last accessed on February 14, 2018). False-positive variants were curated manually using the Integrative Genomics Viewer [23]. Because CCOs comprise pure cancer epithelial cells, variants with a variant allele fraction (VAF) > 0.9 were considered to be biallelic.

### Analysis of copy number variation

To determine copy number variation (CNV), the organoid DNA bam file was analyzed using CNVkit (v0.9.0) [24]. In this study, the CNV fraction was defined as the ratio of the region in which the log2 segmentation value is > 0.4 or the log2 segmentation value is < − 0.4 to the region in the exomes. Copy segments were visualized using the copy number package [25].

### Whole transcriptome sequencing and data processing

Total RNA sequencing was performed for 87 CCOs and matched FFPE primary tumor tissues. For FFPE tissues, manual microdissection of unstained tissue sections was performed for viable tumor areas under a light microscope, using the corresponding H&E slide as a reference. Total RNA was extracted using the RNeasy Mini Kit (Qiagen) and a cDNA library was constructed using the TruSeq RNA Access Library Prep Kit (Illumina), starting with 1 μg of total RNA. All samples passed the cDNA library quality assurance tests (minimum requirement > 5 nM). Paired-end sequencing of 100 nt fragments was performed on the HiSeq 2500 Illumina platform. RNA sequence data from primary cancer tissues and CCOs were processed in a similar manner; raw RNAseq data were processed using the TCGA RNAseq Pipeline (v2) after sequence quality assurance, and the quality of the FastQ files was checked using FastQC (https://github.com/s-andrews/FastQC, v0.7.15). The FastQC “basic statistics” results for all primary cancer tissues and CCOs were “PASS”. Primary cancer tissues had the following average basic statistics: total sequence, 50,391,531.95; GC, 47.97%; total deduplicated percentage, 23.98. The organoids had the following average basic statistics: total sequence, 43,461,472.52; GC, 50.18%; total deduplicated percentage, 19.85. TCGA RNAseq Pipeline (v2) was used to analyze raw RNAseq reads and to quantify gene expression. Reads that passed the quality check were mapped to the human reference genome (hg19) using MapSplice [26] (v2.2.1). RSEM [27] (v1.3.0) was used for transcript quantification. Quantified gene expression was normalized using a fixed upper quartile normalization method. Normalized raw gene expression data for both CCOs and primary tumor tissues have been deposited in Gene Expression Omnibus (GEO; https://www.ncbi.nlm.nih.gov/geo; accession no. GSE171682).

### TIM classification

The immune and stroma classes from the 87 primary cancer tissues were distinguished using the Nearest Template Prediction module [28] implemented in GenePattern (www.broadinstitute.org/genepattern). Each sample was predictively assigned to an “Immunogenic” or “Non-immunogenic” class based on its immune characteristics [1]. The immunogenic class was further divided into a “Normal stroma” and an “Activated stroma” class based on stroma characteristics [2]. Finally, the immunogenic class with normal stroma was classified as immuno-active (Active), the immunogenic class with activated stroma was classified as immuno-exhausted (Exhausted), and the “Non-immunogenic” class was classified as immuno-desert (Desert) [1–3].

### Profiling of tumor-infiltrating immune cells

To identify infiltrating immune cells, CIBERSORT [29] with LM22 (22 immune cell types) gene signatures was used to analyze RNA expression profiles of the 87 primary colon cancer samples or CCOs. The sum of the scores for the 22 cell types was used as the total immune score [11]. MCPcounter [30] was used to identify 10 cell populations in the TIM.

### Cancer signaling pathway analysis

Gene Set Enrichment Analysis (GSEA) v4.0.2 [31] was used to identify alterations in cancer signaling pathways based on RNA expression profiles in the primary tissues or CCOs. The hallmark gene set v7.0 was used as the Molecular Signatures Database, and the GSEA results were considered significant when the FDR q value was < 0.25 and the nominal *p*-value was < 0.05. Gene Set Variation Analysis (GSVA) [32] was used to score the activation of hallmark gene sets and to calculate the CD8^+^ T cell exhaustion score using the CD8^+^ T cell exhaustion gene set [33] for each sample.

### Detection of gene fusions

STAR-Fusion [34] v1.8.1 (default options) was used to detect gene fusions in CCO RNA sequence data. To filter out false-positive results, fusion fragments per million (FFPM) > 0.15 of total RNAseq reads was applied to the STAR-Fusion results. The results were further filtered based on the number of split reads and spanning reads: without any split reads, there should be at least 20 spanning reads, and one split read requires at least 10 spanning reads. When there are more than two split reads, five or more spanning reads are required. False positives were removed manually when the same fusion was detected multiple times due to mis-mapping. The filtered fusions were visualized as circos plots using chimeraviz [35] v1.8.5 in R.

### Variant calling in RNA sequence data

RNA sequence reads were mapped against the human reference genome (National Center for Biotechnology Information build 37) using STAR (2.7.3) [36]. Picard Markduplicates, GATK IndelRealigner, and BaseRecalibrator were implemented in the same way as described above for DNA reads. GATK HaplotypeCaller (3.8.0) and GATK Mutect2 (4.0.2) were used to identify mutations in the RNA BAM files. Mutations discovered using HaplotypeCaller were filtered based on the following criteria: FS > 30.0 and QD < 2.0. For variants detected by Mutect2, only the “PASS” from FilterMutectCalls was used. This analysis pipeline can also accurately detect variants in RNA sequences [37]. RNA variants were identified, and only those variants found in CCO samples were analyzed. Sequencing depth at the mutated position was calculated using the “depth” option in samtools.

### HLA typing

Seq2HLA [38] (v2.3) was used to identify high-resolution HLA molecular types from organoid RNA sequence data. HLA class I alleles were identified with four-digit resolution. HLA-A and HLA-B results (including the ambiguity flag) were mapped to the HLA supertype [39].

### Gain-of-function (GOF) mutations in *TP53*

GOF mutations in *TP53* variants were classified based on previously described criteria [40]. Briefly, 103 p53 mutant proteins were evaluated based on three categories of GOF activity, i.e., 1) interference with p73 activity, 2) transactivation of genes downregulated by wild-type p53, and 3) cooperation with oncogenes during transformation of rat or mouse embryonic fibroblasts [41]. Based on these criteria, 31 p53 mutations (S127Y, P151S, R156P, Y163N, Y163C, V173L, R175H, C176Y, H179R, H179Q, L194R, Y205C, H214R, Y220C, Y234C, M237I, S241F, G245C, G245S, G245V, G245D, R248W, R248G, R248Q, R273C, R273L, R273H, R273P, C275Y, D281G, and R282W) were classified as GOF mutations [40]. The remaining p53 mutations were classified as having no evidence of GOF activity (NE-GOF).

### Molecular classification based on whole transcriptome data

To identify cancer subgroups with specific signaling pathways, all CCO samples in hallmark v7 gene sets were scored using GSVA [32], based on CCO mRNA expression. Subgroups were identified using k-means clustering analysis and principal component analysis (PCA). The results were visualized using the R package ComplexHeatmap [42]. Consensus Molecular Subtype (CMS) classification of organoid transcriptome data was performed in R using the CMScaller package, which is suitable for colorectal cancer pre-clinical models [43].

### Microsatellite instability (MSI) testing

MSI status was evaluated using polymerase chain reaction (PCR). Fluorescence-labelled primers were used to amplify five microsatellite loci, two mononucleotide repeats (BAT-25 and BAT-26), and three dinucleotide repeats (D5S346, D2S123, and D17S250) in tumors and matched normal samples. MSI status was based on the length of the PCR product within the tumor sample versus in the paired normal sample. Samples with instability in two or more of the five loci were defined as microsatellite instability-high (MSI-H), samples with instability in one of the five loci were defined as microsatellite instability-low (MSI-L) and samples with no instability were defined as microsatellite stable (MSS). Two samples were excluded from MSI-PCR testing and status was determined using our previously reported algorithm based on targeted DNA sequence data [44, 45].

### Enrichment analysis

The enrichment score (ES) was calculated to determine whether samples with specific binary events were enriched in certain targets with continuous variables across the whole sample [46, 47]. The 87 CCOs with corresponding CD8^+^ T cell immune scores were ordered based on increasing immune scores. Next, the enrichment score for biallelic alterations identified in *KRAS* in the CCOs was calculated based on the ordered immune scores and then normalized using the Kolmogorov–Smirnov statistics [46, 47], as shown below. The enrichment score for *KRAS* biallelic alterations (KBA) in the organoids was calculated as follows:

$$ \mathit{\operatorname{MAX}}\left({\sum}_{j=1}^n\sqrt{\frac{N-G}{G}}\ \left( if\ sample\ j\  in\  KBA\right)-{\sum}_{j=1}^n\sqrt{\frac{G}{N-G}}\ \left( if\ not\ sample\ j\  in\  KBA\right)\right)\#(1) $$

N = total organoid sample number; G = number of organoid samples with KBA.

The enrichment score had a higher positive score when samples with KBA were consistently ranked at the top of the list of whole samples. The maximum enrichment score was obtained when the n^th^ sample in the KBA was ranked as the top enrichment score among whole samples. The KBA class label was permutated 10,000 times, and the maximum enrichment score generating background distribution was recorded each time. The permutated *P*-value was then calculated as follows:

$$ \overset{\sim }{p}={B}^{-1}\sum \limits_{b=1}^BI\left({maxES}_0\le {maxES}_b\right),B=\mathrm{10,000}\#(2) $$

The ES for *TP53* GOF mutations was calculated in the same manner.

### Analysis of public cancer cell line genomics data and differential dependency

Cancer cell line genomic data, including mutation calls, were downloaded from the DepMap 19Q4 data release (https://depmap.org/portal) [48]. Annotation of primary disease sites for each cell line can be found in the DepMap 19Q4 data release. Genes with dependencies in cell lines with the *FBXW7* mutation (hotspot or damaging mutation) were identified and compared with those in cell lines without the *FBXW7* mutation. Differences in mean dependency between *FBXW7* mutated and non-mutated cell lines were estimated, and the associated *P*-values were computed using the Wilcoxon rank-sum test. In addition, sensitivity of 1001 molecularly annotated human cancer cell lines to 265 drugs was assessed [49]. Cell line mutation data were obtained from the Cell Lines Project v91 (https://cancer.sanger.ac.uk/cosmic).

### Analysis of publicly available single cell RNA sequence (scRNAseq) data

Normalized scRNAseq data from 17,469 epithelial cells from 23 colon cancer tissues were downloaded from the Gene Expression Omnibus database (https://www.ncbi.nlm.nih.gov/geo/; accession no. GSE132465) [50]. The Rtsne v0.15 package in R was used for clustering and visualization of the scRNAseq expression data.

### Analysis of publicly available CCO and TCGA data

Normalized RNA sequence data for CCOs were downloaded from the Gene Expression Omnibus database (https://www.ncbi.nlm.nih.gov/geo/; accession no. GSE65253) [11]. Level 3 RNAseq gene expression data (released February 4, 2015) (illuminahiseq rnaseqv2-RSEM_genes_normalized) for colorectal cancer were downloaded from TCGA and pre-processed at the Broad Institute (https://gdac.broadinstitute.org). Clinical data were downloaded from cBioPortal (https://www.cbioportal.org/).

### Drug testing in CCOs

CCOs were cultured in 24-well plates for 2 weeks, and then harvested and dissociated using TrypLE Express. The dissociated CCOs were mixed with MBM + Matrigel (1:3 ratio) and seeded in 96-well white plates (10 μL of 2 × 10^3^ cells/well; Corning). After gelation, 100 μL MBM was added to each well. The CCOs were allowed to grow for 10–14 days until their diameters reached 100 μm. Then, ten concentrations of FH535, ICG-001 (all Selleckchem, TX, USA), and DMSO controls were added every 3 days. After 6 days, the medium was changed to 100 μL MBM per well to measure cell viability, and 100 μL CellTiter-Glo (Promega) was added to each well. The experiments were conducted in triplicate. The plates were agitated for 30 min at room temperature prior to luminescence measurement. IC_50_ values were determined using Graph Pad Prism (v5.01).

### Statistical analysis

Continuous variables were analyzed using Spearman’s correlation analysis. Depending on the normality of data distribution, the Wilcoxon rank-sum test (non-parametric) or Student’s *t*-test (parametric) was used to evaluate the significance of differences in continuous variables between groups. Fisher’s exact test or the chi-square test was used to evaluate the significance of the differences between categorical variables. Multivariate Cox proportional hazards regression and logistic regression analyses were also performed. All statistical analyses were performed in R version 3.5.2.

## Results

### Development of CCOs and genomic characterization

**Fig. 1:**
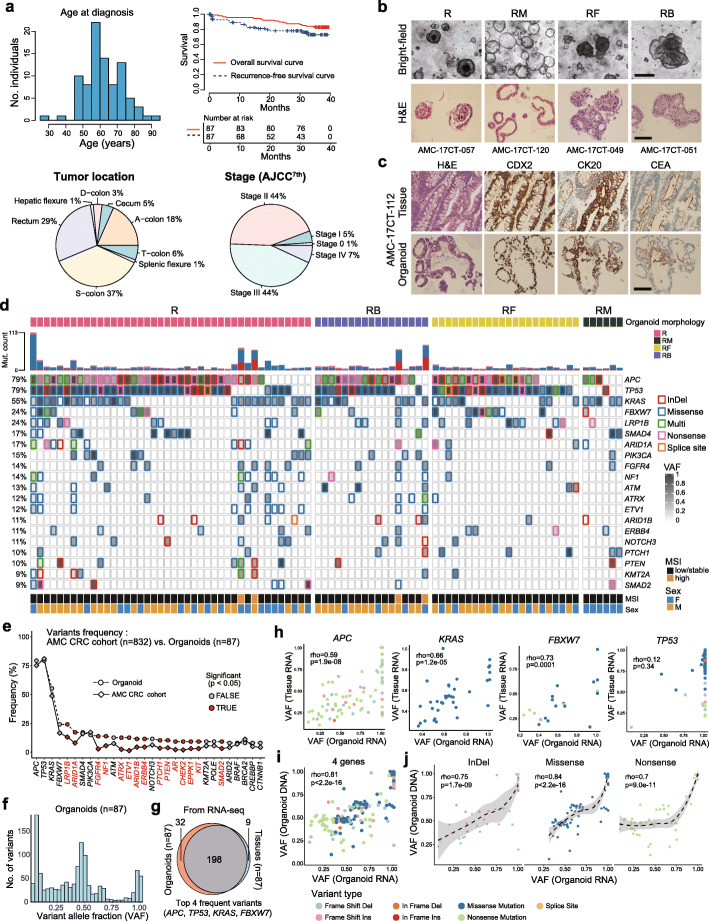
**Characteristics of 87 CCOs. (A)** Clinical features, including age, survival, tumor location, and cancer stage of the 87 patients with colorectal cancer. **(B)** Histology of the CCOs with 4 morphology types (R, thick-walled spherical; RM, thin-walled bubble-like; RF, compact globular; RB, budding tubular). **(C)** Immunohistochemical findings of CCOs that recapitulate their primary tumors. **(D)** Mutation profiles of the 87 CCOs. **(E)** Comparison of mutation frequencies between the 87 CCOs and an independent set of 832 colorectal cancers. **(F)** Diverse distribution of VAF in the 87 CCOs. **(G)** Overlap between the variants in CCOs and their primary tumors based on the top 4 most frequently mutated genes. **(H)** Spearman’s correlation coefficients in VAFs in each gene in organoids and primary tumors (Spearman’s correlation test). **(I)** Significant correlation of the VAFs in the top 4 most frequently mutated genes between DNA and RNA from the 87 CCOs (Spearman’s correlation test). **(J)** Significant correlation of the VAFs in top 4 most frequently mutated genes between DNA and RNA from the CCOs according to variant type (Spearman’s correlation test). CCO, colorectal cancer organoid; H&E, hematoxylin and eosin; VAF, variant allele fraction; MSI, microsatellite instability

A total of 87 patient-derived CCOs, cultured from resected colorectal cancer tissues (Fig. 1a **and Supplementary Table** **1**) as part of the cancer organoid biobank project, were used. CCOs showed diverse morphologies under a bright-field microscope, including thick-walled spherical (R), thin-walled bubble-like (RM), compact globular (RF), and budding tubular (RB) shapes (Fig. 1b). H&E staining revealed that CCOs maintained the characteristic histology of their respective primary cancer. IHC showed that the CCOs recapitulate the expression patterns and percentages of positive cells for transcription factor CDX2, differentiation marker cytokeratin20 (CK20), and carcinoembryonic antigen (CEA); these markers were positive in most cancer cells (Fig. 1c **and Supplementary Fig.** **1****a**). Histological examination revealed that tumor organoids comprised only tumor cells, with no TIM components, such as lymphocytes, fibroblasts, and blood vessel components.

The top 20 mutated genes in the CCOs are shown in Fig. 1d. Although mutations in all genes could not be identified because mutation analysis was based on targeted sequencing, the most frequently mutated genes were *APC* and *TP53* (in 79% of the CCOs), followed by *KRAS* (in 55% of the CCOs), and *FBXW7* (in 24% of the CCOs). These frequencies were similar to those previously reported for colorectal cancer [51]. The frequencies of these known major driver gene mutations were similar to those observed in an independent colorectal cancer cohort at Asan Medical Center (*n* = 832), which were examined using the same targeted NGS platform. However, other genes showed higher mutation frequencies in CCOs than in the cancer cohort (Fig. 1e). The higher rate of detection of mutations in CCOs is likely due to the high tumor cell content. Additionally, the genetic variants mainly arose as subclonal events. In fact, the CCOs had widely distributed VAFs (Fig. 1f **and Supplementary Fig.** **1****b)**, indicating the presence of subclones. These data suggest that organoids have intra-tumoral heterogeneity, as previously shown [8].

We further examined correlations in VAF between CCOs and primary tumors using RNA sequences of the top 4 most frequent mutations, which mostly overlapped between CCOs and primary tumors (Fig. 1g-h). Interestingly, we observed a significant tendency toward loss-of-heterozygosity (LOH) in *TP53* (Fig. 1h**)**. This is consistent with a previous report showing that more than 91% of the *TP53* mutations undergo biallelic inactivation due to biallelic mutations or copy-neutral LOH at the DNA and RNA levels [52]. We also observed significant correlations with respect to VAFs between organoid RNA and organoid DNA (Fig. 1i **and Supplementary Fig.** **2****a**), as well as between organoid DNA and tissue RNA (**Supplementary Fig.** **2****b-d**). With respect to correlations between DNA and RNA, we observed non-linear patterns, especially in the case of nonsense mutations (Fig. 1j); this may be explained by nonsense-mediated decay of mRNA [53]. Various copy number alterations were also present across CCOs. Organoids with MSI-H exhibited subtle copy number alterations (**Supplementary Fig.** **3****a**). A total of 36 fusions were identified in 25 CCOs (**Supplementary Fig.** **3****b**). However, no known targetable fusion events were present. The frequency of HLA class I molecular types (**Supplementary Fig.** **3****c-d**) was in good agreement with that previously reported in 5082 Koreans [54].

### Oncogenic gene expression signatures are maintained in organoids

**Fig. 2:**
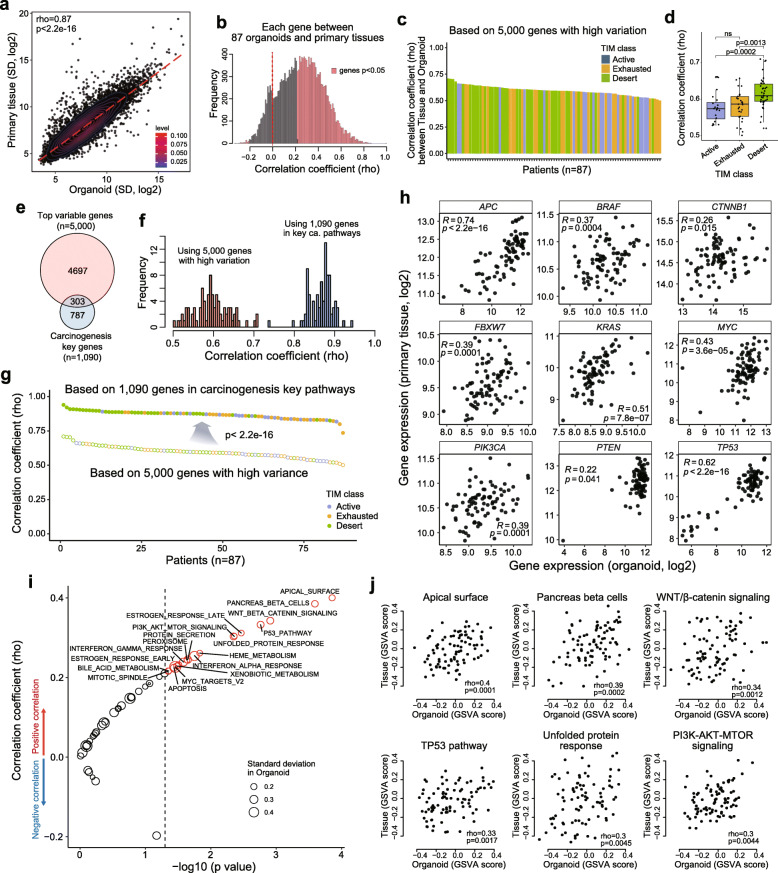
**Correlation in gene expression between CCOs and primary tumor tissues.** Correlation of the expression of 12,114 genes expressed in CCOs and primary tissues from 87 patients (Spearman’s correlation test) **(A)** and frequency of the correlation coefficients **(B)**. Correlation coefficients for 5000 selected genes and tumor immune microenvironment (TIM) status of primary tumors in 87 patients **(C)** showed higher correlation coefficients in immuno-desert group **(D)** (Wilcoxon rank-sum test). **(E)** Overlap between the top variance genes and key genes involved in carcinogenesis. Spearman’s correlation coefficients between CCOs and primary tissues with respect to the top variance genes and key genes involved in carcinogenesis **(F)** and higher correlation coefficients were noted in key genes of cancer pathways (Wilcoxon rank-sum test) **(G)**. **(H)** Correlation in the expression of individual genes between CCOs and primary tissues (Spearman’s correlation test). Correlations in signaling pathway activities based on GSVA analysis of CCOs and primary tissues (Spearman’s correlation test) **(I)** and representative figures of individual pathways **(J)**. CCO, colorectal cancer organoid

Next, we examined whether oncogenic gene expression signatures of the primary tumors were maintained in the organoids. First, we found that genes exhibiting log2 expression of 5 or higher (*n* = 12,114) among patients were significantly correlated between organoids and primary tissues (Fig. 2a). Most genes had significant positive correlations (Fig. 2b **and Supplementary Fig.** **4****a**). We selected 5000 genes with the highest variance in organoids and calculated the Spearman’s correlation coefficient between organoids and primary tumors. We found significant correlation in mRNA expression between organoid and matched tissues in the 87 patients (correlation coefficients range: 0.501–0.71, Fig. 2c). These correlation coefficients were significantly higher in the Desert group, which was characterized by a low level of immune cell infiltration (Fig. 2d).

After confirming that the global expression patterns of the 5000 genes were similar in organoids and primary tumor tissues, we tested correlations using 1090 key genes involved in carcinogenesis pathways from the hallmark gene sets v7; these genes were involved in Wnt/β-catenin, DNA repair, Notch, PI3K/AKT/mTOR, MYC target, P53, and KRAS signaling pathways. The majority (72%) of these genes showed no overlap with the 5000 genes with high variance (Fig. 2e). A significant correlation was identified between the expression of this gene set in organoids and primary tissues in the 87 patients (correlation coefficient range: 0.736–0.941); the correlation coefficients for this gene set were significantly higher than those for the set of 5000 genes with high variance (Fig. 2f-g). Among the genes showing a significant correlation between organoids and primary tissues (e.g. *APC*, *BRAF*, *CTNNB1*, *FBXW7*, *KRAS*, *MYC*, *PIK3CA*, *PTEN*, and *TP53*), *APC* showed the highest correlation (rho = 0.74, *p* < 2.2e^− 16^; Spearman’s correlation test; Fig. 2h).

We then tested whether cancer signaling pathways were maintained in the organoids. All primary tissues and organoids were scored for the hallmark gene set v7 using GSVA [32]. We found that most cancer signaling pathways tended to show a positive correlation (Fig. 2i) and that there were significant correlations in signaling pathways between organoids and primary tissues, including the WNT/β-catenin, TP53, PI3K/AKT/mTOR, and unfolded protein response signaling pathways (Fig. 2j). In summary, we verified at multiple levels—i.e., total genes, high variance genes, genes involved in key carcinogenesis pathways, important cancer-related genes, and genes involved in cancer signaling pathways—that organoids recapitulate the primary tumors in terms of gene expression and mechanisms of carcinogenesis.

We also found that—as expected—microenvironmental signatures of gene expression were the major factors that distinguished organoids from primary tissues (**Supplementary Fig.** **4****b-c**). However, the expression of differentiation markers or stem cell markers of intestinal epithelium [55, 56] was also correlated between organoids and primary tumor tissues despite the absence of TIM in organoids (**Supplementary Fig.** **4****d).** Taken together, the gene expression patterns in organoids represent tumor cell-specific signatures (separate from the TIM).

### Cancer-intrinsic signaling pathways in CCOs

**Fig. 3:**
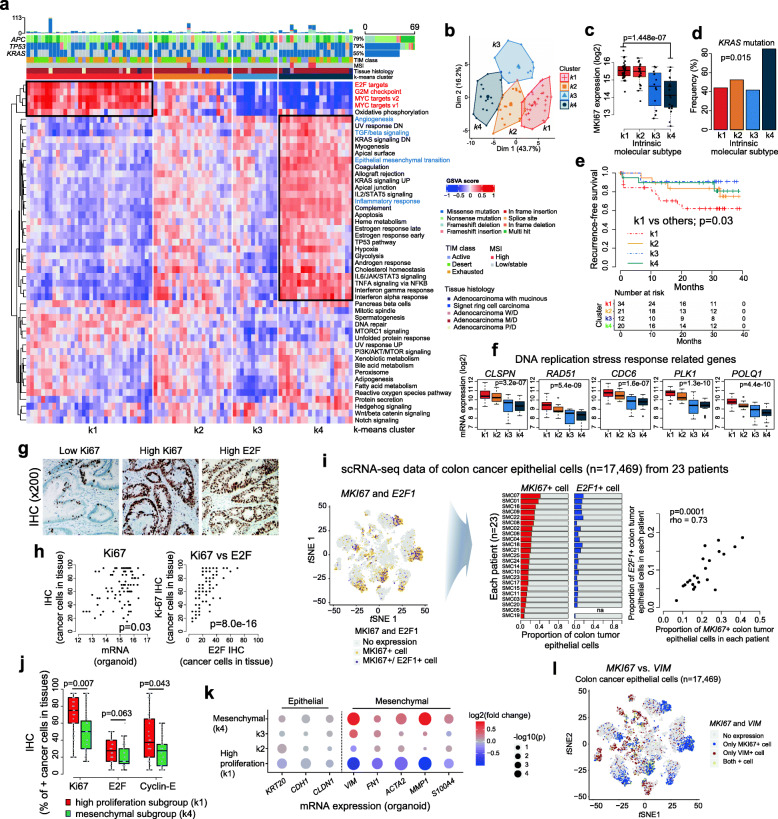
**Cancer-intrinsic signaling pathways in the 87 CCOs.** Clustering **(A)** and principal component analysis **(B)** of signaling pathways in the 87 CCOs identified four subgroups. **(C)** Significantly higher expression of Ki67 mRNA in k1 (high proliferation) subgroup compared to k4 (mesenchymal) subgroup (Wilcoxon rank-sum test). **(D)** Different frequencies of *KRAS* mutation in each subgroup (Fisher’s exact test). **(E)** Recurrence-free survival based on subgroup shows the worst prognosis in k1 subgroup (log-rank test). **(F)** Higher expression of DNA replication stress response-related genes in high proliferation subgroups such as k1 subgroup (Spearman’s correlation test). Immunohistochemical analysis of primary tissues **(G)**: correlation between Ki67 protein expression in cancer cells of primary tumor tissues and Ki67 mRNA expression in organoids, and correlation between Ki67 protein and E2F protein expression in cancer cells of primary tissues (Spearman’s correlation test) **(H)**. **(I)** Expression of Ki67 and *E2F1* in colon cancer epithelial cells at the single cell level (left panel) reveals significant correlation in the percentages of Ki67-expressing cells and *E2F-*expressing cells (right panel) (Spearman correlation test). **(J)** Immunohistochemistry patterns of cell proliferation related markers, such as Ki67 and Cyclin-E, in cancer cells derived from tissues were correlated with molecular subtypes of the high proliferation (k1) and mesenchymal (k4) subgroups in CCOs (Wilcoxon rank-sum test). **(K)** The k4 subgroup shows higher expression of mesenchymal genes compared with the k1 subgroup (Wilcoxon rank-sum test). **(L)** Rare overlap between Ki67-expressing cells and vimentin-expressing cells at the single cell level. CCO, colorectal cancer organoid; scRNAseq, single cell RNA sequencing

Gene expression analysis revealed that variable alterations in cancer signaling pathways were present across all 87 CCOs, indicating inter-tumoral heterogeneity (as expected for tumors from different patients). Unsupervised clustering using k-means (Fig. 3a) and principal component analysis (Fig. 3b) identified four subgroups, two of which were designated the high proliferation subgroup, (k1), in which E2F/MYC pathways were activated, and the mesenchymal subgroup, (k4) (Fig. 3a and c). The k2 and k3 subgroups were in a “grey zone”, with no specific alterations in gene expression associated with particular pathways. The mesenchymal subgroup (k4) harbored frequent *KRAS* mutations (Fig. 3d), and the high proliferation subgroup (k1) showed the worst prognosis in terms of recurrence-free survival (Fig. 3e). The high proliferation subgroup (k1) showed high expression of genes associated with DNA replication stress responses (Fig. 3f), indicating active replication status. IHC was performed in primary tissues to detect Ki67, E2F, and cyclin-E (Fig. 3g **and Supplementary Fig.** **5****a-b**). Ki67 expression in cancer cells derived from these primary tissues (Ki67-IHC) correlated with the expression of Ki67 mRNA in the organoids, although no correlation was identified between Ki67 mRNA expression in organoids and primary tissue containing TIM (Fig. 3h and **Supplementary Fig.** **5****c**). Additionally, the Ki67-IHC correlated with E2F-IHC in cancer cells derived from primary tumor tissues (Fig. 3h **right**). We validated these findings using publicly available scRNAseq data [50] and found that cancer cells expressing Ki-67 and E2F1 overlapped and the percentage of cancer cells expressing Ki-67 varied among the 23 patients and correlated with the percentage of cancer cells expressing E2F1 (Fig. 3i). When the k1 subgroup (high proliferative group) was compared with the k4 subgroup (mesenchymal subgroup), the k1 subgroup in organoids showed IHC features similar to those observed in the corresponding primary tumors (Fig. 3j), suggesting that the high proliferation activities of primary cancers were maintained in the organoids. In addition, at the gene level, increased expression of mesenchymal genes, including vimentin, was identified in the k4 subgroup of CCOs while decreased expression was observed in the k1 subgroup (Fig. 3k). Thus using scRNAseq data, we verified that most of the single cells expressing MKI67 and the cells expressing vimentin do not overlap (Fig. 3l), which suggests that the high proliferation subgroup and mesenchymal subgroup are distinct subgroups in colorectal cancers.

Clustering analysis based on CMS classification [43] of organoids revealed significant correlation (**Supplementary Fig.** **5****d**), suggesting that the intrinsic molecular subtypes could represent the distinct colorectal cancer subgroups.

### Variable cancer-intrinsic expression of immune-related genes in CCOs

**Fig. 4:**
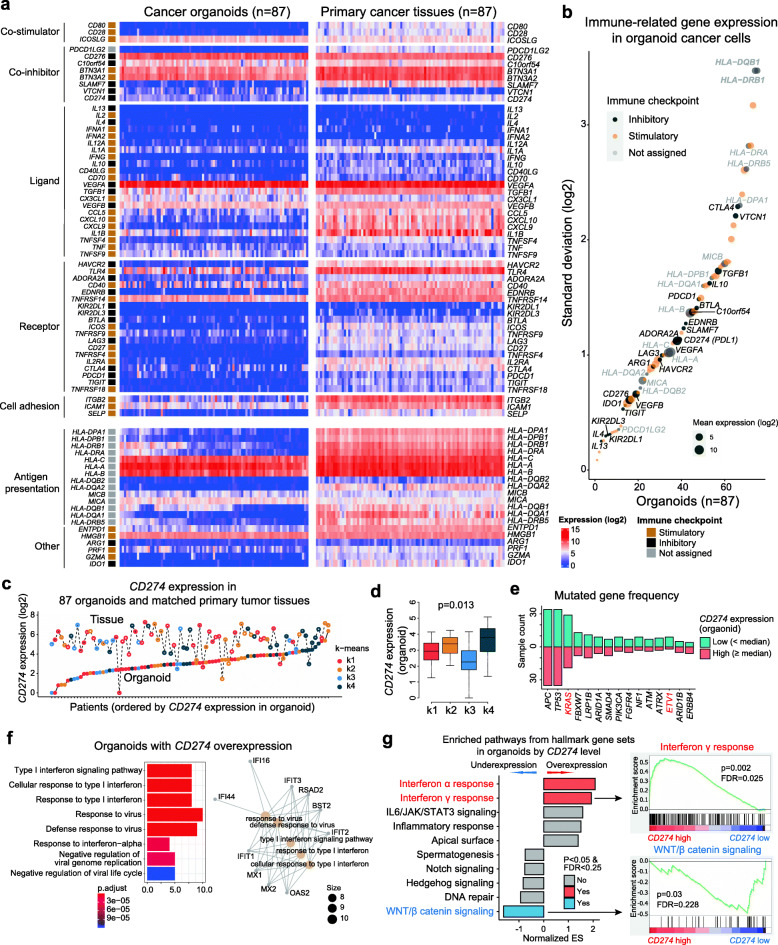
**Expression of cancer-intrinsic immune-related genes. (A)** Expression of immune- and immune checkpoint-related genes by CCOs and primary tissues. **(B)** Variable expression of immune-related genes by CCOs, specifically HLA class II genes. **(C)** *CD274* (PDL1) expression in CCOs and primary tumor tissues. There was no correlation in *CD274* expression between them. **(D)** *CD274* expression based on the molecular subgroups with higher expression in the k4 (mesenchymal) subgroup (Kruskal-Wallis test). **(E)** Frequencies of mutated genes based on the *CD274* expression level of the CCOs. **(F)** Enriched signaling pathways using GO (gene ontology) term database in CCOs with higher *CD274* expression. **(G)** Enriched signaling pathways using hallmark gene set database in CCOs based on *CD274* expression levels. CCO, colorectal cancer organoid

Next, we examined the expression of previously identified immune-related genes [57] (Fig. 4a**).** CCOs expressed various immune-related genes at high levels with a high standard deviation, including HLA class II genes and genes related to immune checkpoint regulation (Fig. 4b). A subset of CCOs overexpressed PDL1 (CD274), independently of total PDL1 expression levels in tissue samples (Fig. 4c). Overall, PDL1 expression was higher in primary tissues than in organoids (**Supplementary Fig.** **6****a**) and tended to be higher in the k4 (mesenchymal) subgroup of organoids (Fig. 4d). Although PDL1 expression in organoids did not correlate with expression in tissues, PDL1 expression in primary tissues correlated with the degree of immune cell infiltration of primary tissues (**Supplementary Fig.** **6****b**). These findings indicate that the main source of PDL1 expression in colon tumor tissues is inflammatory cells. However, PDL1 expression varied among organoids; a subset of organoids exhibited relatively high cancer-specific expression of PDL1. The frequency of individual mutated genes did not differ significantly between the high (≥ median) and low (< median) PDL1 expression groups (Fig. 4e). However, organoids showed different gene expression patterns based on the level of PDL1 expression (**Supplementary Fig.** **6****c**). Gene Ontology (GO) enrichment analysis revealed that CCOs with high PDL1 expression were enriched in GO terms associated with interferon signaling (Fig. 4f). CCOs with high PDL1 expression were found to be enriched in genes associated with interferon α/γ response pathways in a GSEA with 50 hallmark gene sets, whereas CCOs with low PDL1 expression tended to be enriched in genes associated with the Wnt/β-catenin signaling pathway (Fig. 4g). These findings suggest that alteration of pathways intrinsic to cancer cells is associated with PDL1 expression levels and presence of cancer cells with intrinsic alteration of immune related pathway such as interferon signaling.

### Impact of cancer-intrinsic immune-related gene expression on survival

**Fig. 5:**
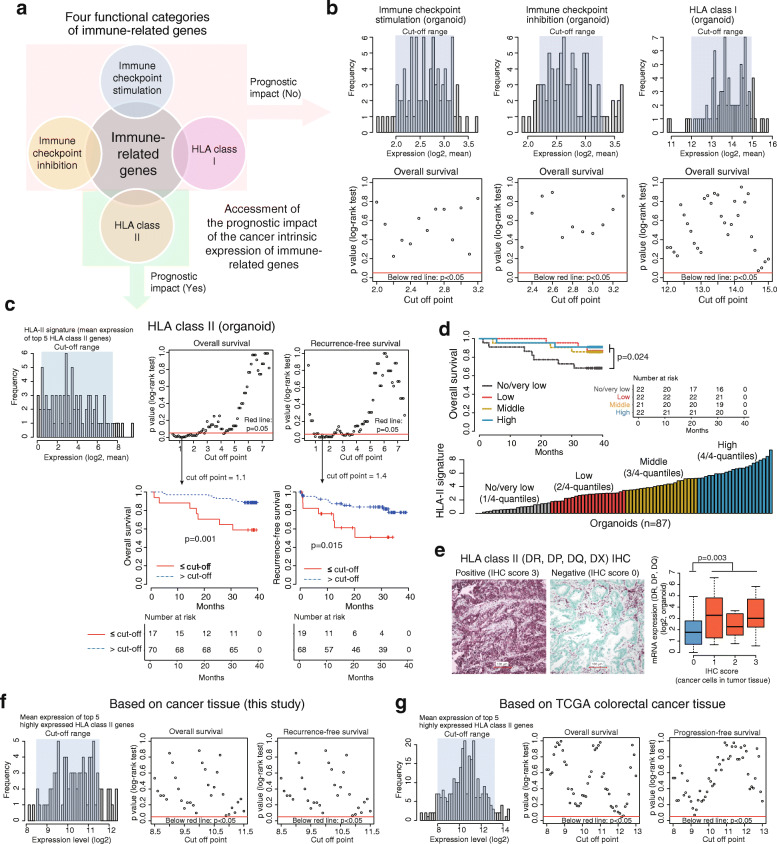
**Functional categories of immune-related genes and patient survival. (A)** Four functional categories of immune-related genes. **(B)** Cancer cell-intrinsic expression of genes associated with immune checkpoint activation/inhibition or HLA class I molecules had no prognostic impact. **(C)** Significant association between cancer-intrinsic expression of HLA class II molecules (DRB1, DQB1, DRA, DRB5, and DPA1) and patient survival. **(D)** Patient’s survival based on 4-quantiles of the HLA-II signatures from CCOs. Patients with no/very low HLA-II signature in CCOs showed unfavorable prognosis, while the other groups showed similarly favorable prognoses. **(E)** Correlation between mRNA expression of HLA class II genes in CCOs and protein expression by IHC in primary tissues (Wilcoxon rank-sum test). No prognostic relevance of the HLA-II signature measured from primary cancer tissues was observed in this dataset **(F)** and in the public TCGA dataset **(G)**. CCO, colorectal cancer organoid; IHC, immunohistochemistry

Next, we classified immune-related genes into four functional categories, i.e., immune checkpoint stimulation, immune checkpoint inhibition, HLA-I, and HLA-II (Fig. 5a). The mean expression values of related genes were used to represent the signature of each functional category. Among them, cancer-intrinsic expression of HLA-I and of immune checkpoint stimulators and inhibitors was not associated with survival in any percentile cut off (Fig. 5b). However, the cancer-intrinsic expression of the HLA-II signature (of the top five highly expressed genes, i.e., DRB1, DQB1, DRA, DRB5, and DPA1, from CCOs), was significantly associated with patient survival (Fig. 5c**).** Assessment of the prognostic impact of the expression of the five individual HLA-II genes (for the top 5 HLA-II genes from CCOs) also showed similar results **(Supplementary Fig.** **7****a**). These findings indicate that cancer-cell intrinsic HLA-II expression has a prognostic impact. We also explored the prognostic impact of HLA-II signature in CCOs based on expression levels using 4-quantiles (Fig. 5d). The CCO patient group with no/very low HLA-II signature showed the worst prognosis. Interestingly, the CCO patient groups with low, medium or high HLA-II signatures showed good prognosis, irrespective of HLA-II signature levels (Fig. 5d). These findings indicate that cancer intrinsic HLA-II expression is associated with favorable prognosis. However, higher intrinsic HLA-II signature level does not mean a better prognosis. Expression of HLA-II DR, DP, and DQ in CCOs correlated with protein expression detected by IHC in cancer cells derived from primary tissues (Fig. 5e). When patients were stratified based on IHC data, patients with higher expression of HLA-II showed better survival, although this was not significant (**Supplementary Fig.** **7****b**). The prognostic impact of HLA-II signature could not be identified upon using primary tissues from two independent sets, i.e., our own (Fig. 5f) and TCGA colorectal cancer data (Fig. 5g); this suggests that cancer-intrinsic expression of HLA-II plays an important role in predicting patient prognosis because bulk cancer tissue contains TIM that includes antigen-presenting cells such as mononuclear inflammatory cells, which are the main centers of HLA-II expression, and the HLA-II signal from those cells adds to the net HLA-II signature in the bulk cancer tissue.

### A subset of CCOs with intrinsic immunogenic properties is associated with improved patient survival

**Fig. 6:**
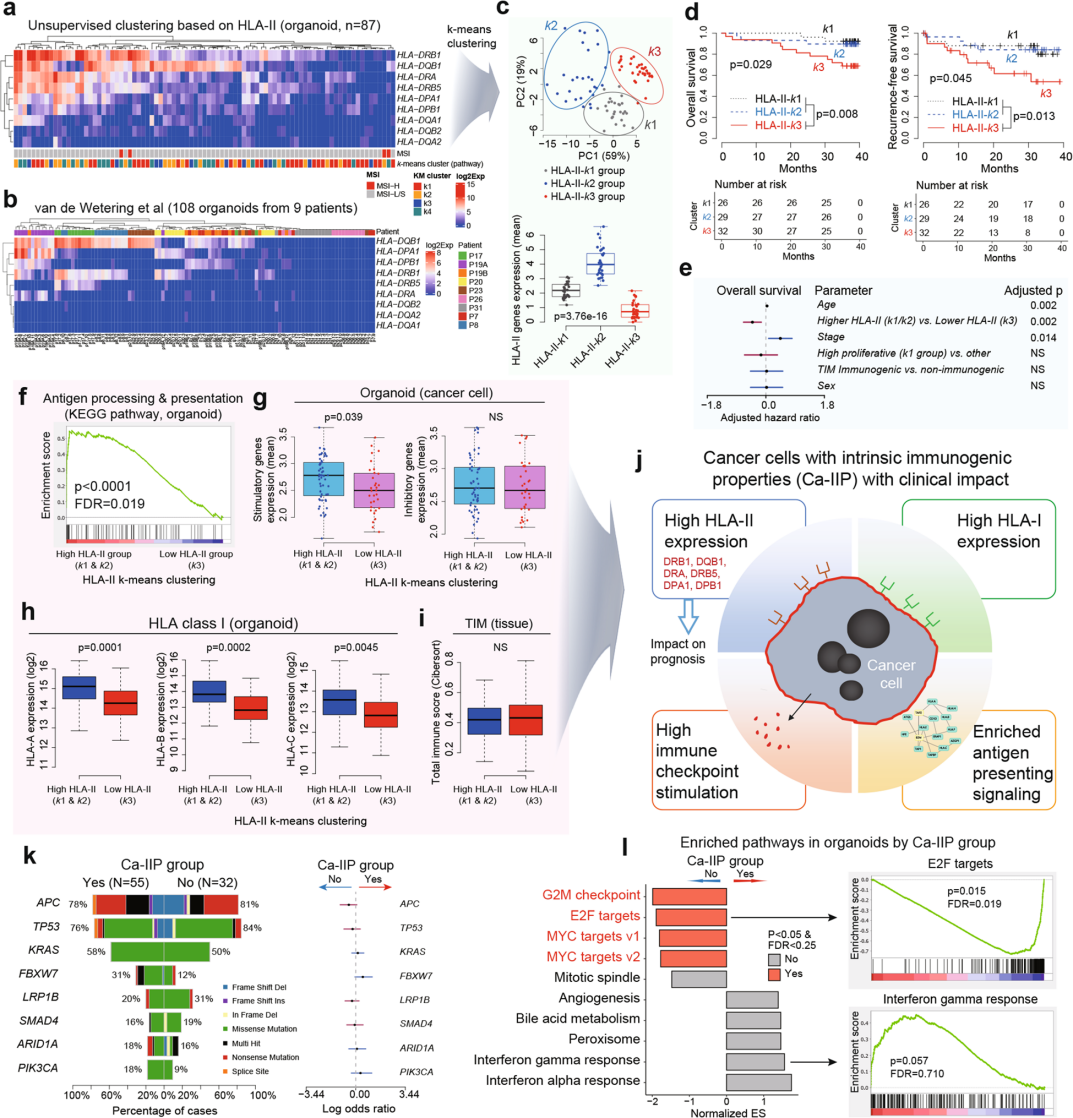
**A subset of CCOs has intrinsic immunogenic properties. (A, B)** Unsupervised clustering based on HLA-II genes expression in two independent CCO cohorts revealed similar clustering patterns. (**C**) k-means clustering based on HLA-II genes expression revealed 3 subgroups, which were correlated with HLA-II gene expression levels (Kruskal-Wallis test). HLA-II-*k*3 subgroup (subgroup with the lowest HLA-II genes expression) resulted in unfavorable prognosis (**D**) and this prognostic impact was independent of patient age, sex, stage, high proliferative molecular subgroup (k1 subgroup) and tumor immune microenvironment (TIM) status (Multivariate Cox regression analysis) (**E**). **(F)** Enriched antigen processing/presentation pathway in CCOs with high HLA-II expression (HLA-II-*k*1 and HLA-II-*k*2 subgroups). CCOs belonging to these subgroups had high intrinsic immune-stimulatory gene expression (**G**) and high HLA class I expression (**H**). However, the total immune cell infiltration amount in primary tissues was not associated with these subgroups (Wilcoxon rank-sum test) (**I**). **(J)** These subgroups (HLA-II-*k*1 and HLA-II-*k*2 subgroups) were referred as Ca-IIP group and their characteristics were summarized. **(K)** There were no significant mutations associated with Ca-IIP (*p* > 0.05 by logistic regression analysis). **(L)** Enriched cancer-intrinsic signaling pathways based on Ca-IIP (GSEA analysis) in CCOs. CCO, colorectal cancer organoid; Ca-IIP, Cancer cells with intrinsic immunogenic properties

Among the immune-related genes expressed by CCOs, *HLA-DQB1* exhibited the most variable expression (Fig. 4b) and appeared to have some clinical impact (**Supplementary Fig.** **7****a**). Therefore, we performed unsupervised clustering based on HLA-II genes, which revealed a tendency to cluster based on HLA-II expression levels, with the highest expression being of that of DRB1, DQB1, DRA, DRB5, DPA1, and DPB1 (Fig. 6a). The clustering pattern in our CCO cohort was similar to that of an independent CCO cohort from nine patients [11] (Fig. 6b). To better stratify the CCOs, we performed k-means clustering and PCA on our CCO cohort and generated three groups, i.e., high HLA-II expression group (HLA-II-*k*2 group), medium HLA-II expression group (HLA-II-*k*1 group), and no/low HLA-II expression group (HLA-II-*k*3 group) (Fig. 6c). The patients in the HLA-II-k3 group had the worst prognosis, whereas those in the HLA-II-k1 group and HLA-II-k2 groups were predicted to have favorable survival (Fig. 6d). This finding is similar to our finding in Fig. 5d. Therefore, HLA-II-k1 group and HLA-II-k2 group were integrated into a single group classified as Higher HLA-II group while HLA-II-k3 group was classified as Lower HLA-II group for further analysis. The prognostic impact of the Higher HLA-II group was independent of other clinicopathological parameters, such as disease stage, age, intrinsic molecular subgroup (k1 subgroup), and immunogenic TIM (Fig. 6e).

To better characterize the Higher HLA-II group, KEGG pathway analysis was performed on the CCO data. The results revealed that Higher HLA-II CCOs had significant enrichment of intrinsic antigen processing and presentation pathways (Fig. 6f), higher expression of signatures associated with intrinsic immune checkpoint stimulation (Fig. 6g), and higher expression of intrinsic HLA-I (Fig. 6h), despite the fact that the Higher HLA-II group was identified based on clustering of HLA-II expression patterns. This group was not associated with the total number of immune cells infiltration in the TIM of primary tumor tissue (Fig. 6i **and Supplementary Fig.** **8****a**). Thus, we refer to the Higher HLA-II group as cancer cells with intrinsic immunogenic properties (Ca-IIP) that have a clinical impact (Fig. 6j). To explore underlying molecular characteristics of Ca-IIP, we compared mutational profiles and gene set enrichment analysis (GSEA) in a hallmark gene set. There were no significant differences in the mutational profiles of CCOs with and without Ca-IIP (Fig. 6k). However, significantly enriched cell-cycle regulatory pathways, including MYC and E2F targets for CCOs with no Ca-IIP, were observed (Fig. 6l). The HLA-I supertype was not associated with Ca-IIP (**Supplementary Fig.** **8****b**).

### Cancer-intrinsic immuno-genomic alterations associated with TIM phenotypes

**Fig. 7:**
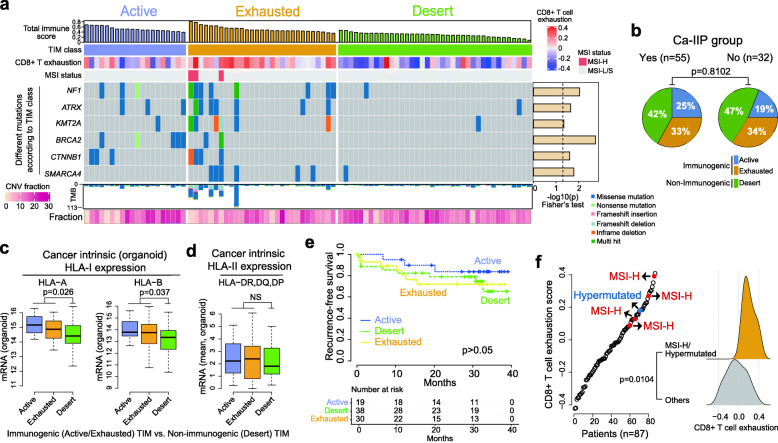
**Characteristics of TIM of primary tissue. (A)** Classification of the TIM in 87 colorectal cancer samples. **(B)** No association between TIM status and Ca-IIP group (Fisher exact test). The TIM status was associated with intrinsic HLA-I expression (**C**) but not intrinsic HLA-II expression (**D**) in CCOs (Wilcoxon-rank sum test). **(E)** Patient survival based on TIM status (log-rank test). **(F)** High CD8^+^ T cell exhaustion score in MSI-H/hypermutated tumors (Wilcoxon-rank sum test). TIM, tumor immune microenvironment; CCO, colorectal cancer organoid; Ca-IIP, Cancer cells with intrinsic immunogenic properties; MSI-H, microsatellite instability high

To identify cancer cell-intrinsic immuno-genomic alterations associated with the TIM, we classified TIM status as immunogenic or non-immunogenic based on the gene expression profiles of the 87 primary tumor tissues and known expression patterns of marker genes; thus the immunogenic TIM was divided into two additional subtypes, i.e., Exhausted and Active (Fig. 7a and **Supplementary Fig.** **9****a)**. The TIM status was validated using different methods, including CIBERSORT immune cell profiling (**Supplementary Fig.** **9****b**), MCP immune and stromal scores (**Supplementary Fig.** **9****c**), and the CD8^+^ T exhaustion score (as measured by the GSVA algorithm) (**Supplementary Fig.** **9****d**). TIM status was not associated with Ca-IIP (Fig. 7b). Additionally, TIM status was significantly associated with cancer cell-intrinsic expression of HLA-I (Fig. 7c **and Supplementary Fig.** **9****e**) but not of HLA-II (Fig. 7d). Clinically, patients with the active TIM had better survival than the other groups, albeit without statistical significance (Fig. 7e). Based on the TIM status, all four tumors with MSI-H and one hypermutated tumor were classified in the Exhausted group (Fig. 7a). These tumors also had high CD8^+^ T exhaustion scores (Fig. 7f). With respect to nonsynonymous mutations, there were differences in the frequency of *NF1* and remodeling genes *SMARCA4*, *KMT2A*, and *ATRX* based on TIM class (Fig. 7a). There were no significant differences in CNV (Fig. 7a **and Supplementary Fig.** **9****f**) or fusion number (**Supplementary Fig.** **9****g**) based on TIM status.

**Fig. 8:**
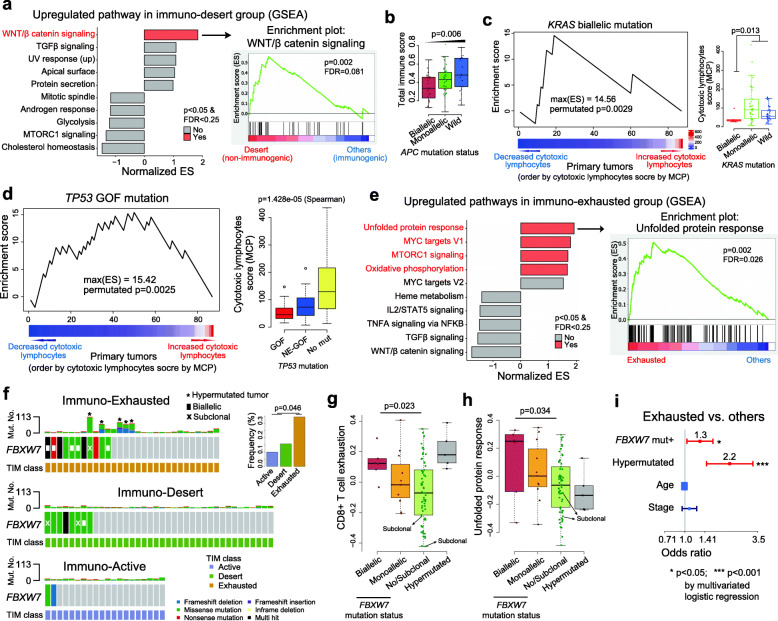
**Cancer intrinsic molecular alterations and the TIM. (A)** Upregulated WNT/beta catenin pathways in CCOs with an immuno-desert microenvironment of primary tumor. (**B**) Correlation between the total immune score (CIBERSORT) and *APC* mutation status (Spearman’s correlation test). **(C)** The *KRAS* mutation is enriched in tumors with low cytotoxic lymphocyte infiltration (10,000 permutated test and Wilcoxon rank-sum test one-sided). (**D**) Association between *TP53* GOF mutations and decreased cytotoxic lymphocyte infiltration (10,000 random permutation test and Spearman’s correlation test). **(E)** Upregulated cancer-intrinsic pathways in CCOs with exhausted TIM of primary tumor (GSEA analysis). **(F)** Frequent *FBXW7* mutation in CCOs with exhausted TIM of primary tumor (Fisher’s exact test). CD8^+^ T cell exhaustion score (**G**) and unfolded protein response pathway score (**H**) by GSVA analysis based on *FBXW7* mutation status (Spearman’s correlation test). **(I)** *FBXW7* mutation and hypermutated phenotype including MSI-H were independently associated with exhausted TIM of primary tumor **(**multivariate logistic regression analysis). GOF, gain-of-function; TIM, tumor immune microenvironment; CCO, colorectal cancer organoid. MSI-H, microsatellite instability-high

Next, we examined genes that were differentially expressed in the different TIM subgroups (Active, Exhausted, and Desert) and found no significant differences at the single gene level among the TIM subgroups in the organoids (one-way ANOVA test, FDR > 0.25 for all tested genes) (**Supplementary Fig.** **10**). Therefore, we focused on signaling pathways and found that the Wnt/β-catenin signaling pathway was enriched in the Desert (non-immunogenic) group (Fig. 8a **and Supplementary Fig.** **11****a-c**). The mutation status of *APC*, a major component of the Wnt/β-catenin signaling pathway, was associated with the Desert group (Fig. 8b **and Supplementary Fig.** **11****d-f**). The *KRAS* (biallelic) mutation was more common in tumors with decreased numbers of cytotoxic lymphocytes (as determined by MCP) (Fig. 8c) or CD8^+^ T cells (as determined by CIBERSORT) (**Supplementary Fig.** **12****a**). These biallelic *KRAS* mutations tended to occur in non-immunogenic cancers (**Supplementary Fig.** **12****b-d**). Colon cancers with the *KRAS* mutation showed upregulated expression of *CXCL3* and *PD-L1* in cancer cells, decreased granzyme B levels in cancer tissues, and increased M2 macrophage numbers in the TIM (**Supplementary Fig.** **12****e**), supporting previous findings from a report of a model showing the characteristics of cancers with *KRAS* mutations [58]. Similarly, the *TP53* GOF mutations were enriched in tumors with low CD8^+^ T cell infiltration (Fig. 8d and **Supplementary Fig.** **13****a-c**). The unfolded protein response pathway was enriched in the Exhausted group (Fig. 8e **and Supplementary Fig.** **13****d**). We also found that the *FBXW7* mutation was more common in the Exhausted group (Fig. 8f), and that it correlated significantly with CD8^+^ T cell exhaustion (Fig. 8g) and the unfolded protein response pathway, based on GSVA (Fig. 8h). Multivariate logistic regression analysis revealed that the association between immuno-exhaustion and *FBWX7* mutation was independent of the hypermutated cancer phenotype including MSI-H (Fig. 8i). The *FBXW7* mutation was one of the most common mutations associated with exhausted TIM (Fig. 8f) and enrichment of genes associated with the unfolded protein response pathway (Fig. 8). Therefore, we screened the Achilles project dataset to identify candidate targets of synthetic lethality in *FBXW7*-mutated tumors. We found that *CTNNB1* inactivation is a potential target in tumors with the *FBXW7* mutation (**Supplementary Fig.** **14****a**). Next, we selected compounds associated with the Wnt/β-catenin pathway and used CCLE data to analyze responses to approximately 200 drugs. We found that cancer cell lines harboring the *FBXW7* mutation had significantly higher sensitivity to the PPAR inhibitor, FH535, than cell lines without the mutation (**Supplementary Fig.** **14****b**). Furthermore, only the colorectal cancer type yielded significant results in the synthetic lethality model (**Supplementary Fig.** **14****c**). We validated the induction of lethality as the PPAR inhibitor for the *FBXW7* mutation using our CCOs (**Supplementary Fig.** **15**), thereby demonstrating the utility of CCOs as a preclinical model.

## Discussion

Tumors with abundant infiltrating T cells are referred to as “immunogenic” or “hot”; these tumors are associated with a favorable prognosis and better response to immune checkpoint inhibitor-based therapy [59, 60]. Therefore, many studies have focused on the TIM. However, the lack of a patient-derived cancer cell model that recapitulates the primary cancer means that relatively few studies have investigated the intrinsic immunogenic properties of cancer cells and their clinical significance. Several studies have shown that CCOs maintain the genetic diversity and treatment response characteristics of the primary tumor [12–15]. Our study revealed that CCOs recapitulate the global gene expression profiles and pathways that characterize the tumors. Although a limited number of genes were evaluated, we confirmed that mutations in known major driver genes, such as *TP53, KRAS, APC*, and *FBXW7* were maintained in the CCOs. Furthermore, we also investigated cancer-intrinsic immunogenomic characteristics along with clinical features in a large CCO sample set.

Cancer organoids enable the identification of signatures associated with cancer-intrinsic immuno-genomic alterations, which are often masked when using bulk tumor tissue due to the signatures within the TME. In this study, we identified Ca-IIP using a large number of CCOs. Patients with Ca-IIP showed an excellent prognosis with respect to overall and recurrence-free survival, with HLA-II being the key intrinsic factor associated with patient survival. We found significant correlation between the expression of HLA-II mRNA in CCOs and HLA-II protein expression in cancer cells from primary tissues. However, the impact of HLA-II IHC data in primary tumor tissue on survival was not significant; this might be explained by the discrepancy between the measurement strategies used in IHC and RNA sequencing; 1) different antibody targets used for HLA-II, 2) tissue microarray (2 mm^2^ × 2 cores) used for IHC may not represent the entire cancer tissue, 3) low resolution of IHC-based expression measurement (categorical measurement) compared to the continuous RNA sequencing results, and 4) small sample size resulting in low statistical power. Indeed, a previous study that measured the expression of HLA class II molecules using IHC in approximately 1000 colorectal cancer tissue samples has shown that the expression of HLA class II antigens in CRC cells was significantly associated with a favorable clinical course [61]. The results from our study suggest that identification of patients based on cancer-intrinsic expression of HLA-II mRNA is a more powerful predictor of survival than identification based on intrinsic expression of HLA-I, intrinsic immune signatures, intrinsic cancer signaling pathways, or TIM status.

Recently, the TIM has received significant attention due to its potential relationship with clinical outcome and response to immunotherapy. As such, we classified colorectal cancers into three microenvironment groups based on immune cell and stromal signatures in primary tumor tissues. Cellular constituents of the TIM are more likely to be induced by intrinsic genomic alterations in tumor cells than by germline genomic characteristics of the host. We identified several genomic alterations in CCOs that were associated with tumor TIM class. All MSI-H tumors belonged to the Exhausted group, which may be associated with a good response to PD1/PDL1 inhibitors because PD1 is an important marker for CD8^+^ T cell exhaustion. In addition, the Desert group showed activation of Wnt/β-catenin signaling and high expression of Wnt pathway target genes, which is consistent with results from recent studies showing that the activation of Wnt/β-catenin signaling results in immune exclusion [3, 62, 63]. Our data also suggest that cancer-intrinsic genetic alterations, including those in *KRAS*, *TP53*, and *FBXW7,* are linked to TIM status.

## Conclusions

The CCOs examined herein recapitulate the gene expression signatures, genetic mutations, and histopathological features of their respective primary tumors. These CCOs provide valuable data for the intrinsic immuno-genomic classification of colorectal cancers. Studying cancer cells with known intrinsic immunogenic properties will provide unique opportunities for the development of novel therapeutic approaches and optimal selection of patients most likely to benefit from targeted therapy.

## Supplementary Information

**Additional file 1.** Supplementary Table 1. Characteristics of patients.

**Additional file 2 Supplementary Fig. 1.** (A) Histology and immunohistochemistry of the established CCOs and the corresponding primary tumors. (B) Distribution of variant allele fraction (VAF) of detected variants in the MSI-high group and the MSI-low/MSS group. CCO, colorectal cancer organoid; MSI, microsatellite instability; MSS, microsatellite stable; H&E, hematoxylin and eosin.

**Additional file 3 Supplementary Fig. 2.** (A) Spearman’s correlation of variant allele fractions (VAFs) between organoid RNA and organoid DNA. (B) Characteristics of variants detected in organoid DNA and not in tissue RNA; the variants detected in organoids only were associated with low read depth in primary tissue sequences or subclonal events based on organoid sequencing (Wilcoxon rank-sum test). (C) Spearman’s correlation of VAFs between organoid DNA and tissue RNA. (D) Spearman’s correlation of VAF in each gene between organoid DNA and tissue RNA.

**Additional file 4 Supplementary Fig. 3.** (A) Profiles of copy number variations with microsatellite instability (MSI) information in the 87 CCOs. (B) Circos plot of the detected fusions in the 87 CCOs. (C) HLA class I molecular typing and its frequency in 87 CCOs. (D) Frequency of the HLA supertype in 87 CCOs. CCO, colorectal cancer organoid.

**Additional file 5 Supplementary Fig. 4.** (A) Spearman’s correlation coefficient matrix of CCOs versus primary tumor tissues. (B) Total immune score and stromal score of CCOs versus primary tumor tissues. (C) Genes that are differentially expressed between CCOs and primary tumor tissues. (D) Spearman correlation of the expression of differentiation or stem cell markers in CCOs versus primary tumor tissues. CCO, colorectal cancer organoid; TIM, tumor immune microenvironment.

**Additional file 6 Supplementary Fig. 5.** (A) Ki-67 immunohistochemistry in the primary tumor tissues and its interpretation by a pathologist and by image analysis. (B) Significant correlation of the Ki-67 proliferation index between the pathologist’s result and image analysis. (C) No correlations in Ki-67 mRNA expression between bulk primary tissues including tumor microenvironment and cancer cells and CCOs. (D) Comparison of our molecular subgrouping and CMS classification in CCOs (Chi-squared test). CCO, colorectal cancer organoid.

**Additional file 7 Supplementary Fig. 6.** (A) PDL1 (*CD274*) expression levels in CCOs and primary tumor tissues. (B) PDL1 expression in primary tumor tissue was correlated with the expression of immune cell markers such as CD8A, CD68, and CD4, but was not correlated with PDL1 expression in CCOs (Spearman correlation test). (C) Differentially expressed genes in CCOs based on cancer-intrinsic (organoid) PDL1 expression level. CCO, colorectal cancer organoid; TIM, tumor immune microenvironment.

**Additional file 8 Supplementary Fig. 7.** (A) Prognostic impact of gene expression in HLA class II in CCOs. (B) Patient survival based on HLA class II expression level using immunohistochemistry (IHC) in cancer cells from primary tissue (log-rank test).

**Additional file 9 Supplementary Fig. 8.** (A) Immune cell profiles of CIBERSORT and MCP based on the Ca-IIP group (Wilcoxon rank-sum test). B cells naive (CIBERSORT) score were lower in the Ca-IIP group. However, B lineage based on MCP score was not significant. (B) HLA class I supertype frequency based on CA-IIP group. Ca-IIP, cancer cells with intrinsic immunogenic properties.

**Additional file 10 Supplementary Fig. 9.** (A) Classification of the TIM using primary cancer tissue. (B) Immune cell types from CIBERSORT based on the TIM class (Wilcoxon rank-sum test). (C) Immune cell types using MCP score based on the TIM class (Kruskal Wallis test). (D) Validation of the exhausted TIM class based on CD8+ T cell exhaustion score using GSVA analysis (Wilcoxon rank-sum test). (E) Significant correlation of HLA-I expression in CCOs versus primary tumor tissues (Spearman correlation test). (F) Fraction of copy number variation (CNV) based on the TIM class (Kruskal Wallis test). (G) Fusion number based on the TIM class (Kruskal Wallis test). TIM, tumor immune microenvironment; CCO, colorectal cancer organoid.

**Additional file 11 Supplementary Fig. 10.** No significant differential gene expression in organoids based on the tumor immune microenvironment (TIM) class of primary tissues (FDR q > 0.25 by one-way ANOVA test).

**Additional file 12 Supplementary Fig. 11.** (A) Pathway analysis using tissue RNA sequence data based on the tumor immune microenvironment (TIM) class. (B, C) Significance of Wnt/β-catenin signaling pathway in the non-immunogenic group using GSVA analysis (Wilcoxon rank-sum test). (D) *APC* mutation status based on the TIM class. (E) Significance of biallelic *APC* mutation and TIM class (Fisher’s exact test). (F) Expression of target genes belonging to the Wnt/beta-catenin pathway (Wilcoxon rank-sum test).

**Additional file 13 Supplementary Fig. 12.** (A) Enrichment of *KRAS* (biallelic) mutations in tumors with decreased CD8+ T cells (10,000 randomly permutated *p* values and one-sided Wilcoxon rank sum test). (B) Increased frequency of *KRAS* biallelic mutation in non-immunogenic (immuno-desert TIM) tumors (Fisher’s exact test). (C, D) *KRAS* mutation status and TIM. (E) Characteristics of gene expression and infiltrating immune cell types based on the *KRAS* mutation status (Spearman’s correlation test). TIM, tumor immune microenvironment.

**Additional file 14 Supplementary Fig. 13.** (A) Frequency of *TP53* gain-of-function (GOF) mutation based on the tumor immune microenvironment (TIM) class, showing a high frequency of *TP53* GOF mutations in the Desert group (*p* = 0.033, Fisher’s exact test). (B) Association between *TP53* GOF mutations and decreased CD8+ T cell (10,000 random permutation test and Spearman’s correlation test). (C) Tendency for the exclusive occurrence of *TP53* and *KRAS* mutation. (D) Enriched unfolded protein response pathway (GSVA score) in the immune-exhausted group (Wilcoxon rank-sum test).

**Additional file 15 Supplementary Fig. 14.** (A) Screening of synthetic lethality target of *FBXW7* mutation using the DepMap dataset. (B, C) Sensitivity of the FH535 molecule in colorectal cancer cell lines with *FBXW7* mutation (Wilcoxon rank sum test).

**Additional file 16 Supplementary Fig. 15.** (A) FH535 induced cell death and destroyed the organoid structure in the two CCOs with *FBXW7* mutation (AMC-17CT-019 and AMC-17CT-048). (B) Sensitivity of the FH535 molecule in CCOs with *FBXW7* mutation (Wilcoxon-rank sum test). (C) Sensitivities of various WNT signaling drugs targets showing non-significant responses to drugs targeting WNT pathways in colorectal cancer cells with *FBXW7* mutation. (D) CCOs with *FBXW7* mutation also showed non-significant responses to ICG-001, which also targets the WNT/β-catenin pathway, suggesting that FH535 has a different mechanism action in colorectal cancers harboring the *FBXW7* mutation. CCO, colorectal cancer organoid.

## Data Availability

The datasets generated and/or analyzed during the current study (normalized raw gene expression data for both CCOs and primary tumor tissues) are available on GEO (https://www.ncbi.nlm.nih.gov/geo; accession no. GSE171682) while the clinico-pathological data for the 87 patients are available in Additional file 1 (Supplementary Table 1).

## Abbreviations

TIM: Tumor immune microenvironment
CCO: Colorectal cancer organoid
GOF: Gain-of-function
MSI: Microsatellite instability
VAF: Variant allele fraction
Ca-IIP: Cancer cells with intrinsic immunogenic properties
CNV: Copy number variation
GSEA: Gene Set Enrichment Analysis
GSVA: Gene Set Variation Analysis
ES: Enrichment score
IHC: Immunohistochemistry
