# Supplementary material for: Immuno-genomic classification of colorectal cancer organoids reveals cancer cells with intrinsic immunogenic properties associated with patient survival
Source: J Exp Clin Cancer Res. 2021 Jul 13;40:230. doi: 10.1186/s13046-021-02034-1 (PMC8276416; doi:10.1186/s13046-021-02034-1)
Supplement: Supplementary file 1 — Additional file 1. Supplementary Table 1. Characteristics of patients. [file 13046_2021_2034_MOESM1_ESM.pdf]

Supplementary Table 1. Characteristrics of 87 patients

Sample_ID	Age	Sex	Type	Differentiation	Subtype	Death	OS(day)	Recur	RFS(day)	CCO Morphology	MSI_PCR	Location	MSI_NGS	HLA.II.IHC.score	Ca-IIP	TIM class	k-means cluster
AMC-17CT-004	67	F	Adenocarcinoma	Moderate	Conventional	0	1211	1	995	R_RF	MSS	A-colon	MSI-L/S	0	Yes	Desert	k2
AMC-17CT-008	49	F	Adenocarcinoma	Moderate	Conventional	0	1208	1	474	R	MSS	Rectum	MSI-L/S	0.5	Yes	Exhausted	k2
AMC-17CT-009	69	F	Adenocarcinoma	Moderate	Conventional	0	1208	0	1191	R_B	MSS	D-colon	MSI-L/S	3	No	Active	k1
AMC-17CT-010	54	M	Adenocarcinoma	Moderate	Conventional	0	1207	0	1124	R_B	MSS	Rectum	MSI-L/S	0	Yes	Active	k4
AMC-17CT-011	71	M	Adenocarcinoma	Moderate	Conventional	0	1204	0	966	RF	MSS	S-colon	MSI-L/S	0	Yes	Desert	k4
AMC-17CT-012	56	M	Adenocarcinoma	Moderate	Conventional	0	1203	1	386	R_B	MSS	Rectum	MSI-L/S	0	No	Exhausted	k1
AMC-17CT-018	59	F	Adenocarcinoma	Moderate	Conventional	0	1197	1	571	R	MSS	D-colon	MSI-L/S	0	No	Desert	k1
AMC-17CT-019	61	M	Adenocarcinoma	Moderate	Conventional	0	1196	0	973	RF	MSI-Low	S-colon	MSI-L/S	0	Yes	Exhausted	k1
AMC-17CT-028	76	M	Adenocarcinoma	Moderate	Conventional	1	703	0	0	R	MSS	S-colon	MSI-L/S	0	Yes	Desert	k2
AMC-17CT-031	77	F	Adenocarcinoma	Moderate	Mucious and conventional	1	94	0	0	R	MSS	A-colon	MSI-L/S	0	No	Exhausted	k1
AMC-17CT-032	59	F	Adenocarcinoma	Moderate	Conventional	0	1189	0	1140	R	MSS	Rectum	MSI-L/S	3	Yes	Active	k3
AMC-17CT-033	66	M	Adenocarcinoma	Moderate	Conventional	0	1189	0	1184	R_RF	MSS	Rectum	MSI-L/S	0	No	Desert	k2
AMC-17CT-035	65	F	Adenocarcinoma	Moderate	Conventional	0	1187	0	1048	R_RF	MSS	Cecum	MSI-L/S	2	Yes	Active	k2
AMC-17CT-036	63	M	Adenocarcinoma	Moderate	Conventional	0	1187	0	1030	R_RF	MSS	T-colon	MSI-L/S	2	Yes	Active	k2
AMC-17CT-037	65	M	Adenocarcinoma	Moderate	Conventional	0	1186	0	1180	R	MSS	S-colon	MSI-L/S	0	Yes	Desert	k4
AMC-17CT-039	76	F	Adenocarcinoma	Moderate	Conventional	0	1183	0	589	RF	MSS	T-colon	MSI-L/S	3	No	Active	k4
AMC-17CT-040	75	M	Adenocarcinoma	Poor	Conventional	0	1182	0	1037	R_B	MSS	A-colon	MSI-L/S	0	Yes	Active	k1
AMC-17CT-042	72	F	Adenocarcinoma	Moderate	Conventional	0	1182	0	1178	R	MSS	S-colon	MSI-L/S	0	Yes	Desert	k4
AMC-17CT-043	60	M	Adenocarcinoma	Moderate	Conventional	0	1181	1	29	R	MSS	Rectum	MSI-L/S	1.5	No	Desert	k1
AMC-17CT-044	76	M	Adenocarcinoma	Moderate	Conventional	0	1180	0	1042	R	MSS	A-colon	MSI-L/S	0	No	Desert	k1
AMC-17CT-045	63	M	Adenocarcinoma	Moderate	Conventional	0	1180	0	1069	R	MSS	A-colon	MSI-L/S	1.5	Yes	Desert	k4
AMC-17CT-046	59	F	Adenocarcinoma	Moderate	Conventional	1	1066	1	317	R	MSS	Rectum	MSI-L/S	0	No	Desert	k2
AMC-17CT-047	55	M	Adenocarcinoma	Moderate	Conventional	0	1179	0	993	R_RF	MSS	Rectum	MSI-L/S	3	Yes	Exhausted	k1
AMC-17CT-048	55	F	Adenocarcinoma	Moderate	Conventional	0	1179	0	1130	R	MSS	T-colon	MSI-L/S	3	Yes	Exhausted	k2
AMC-17CT-049	77	F	Adenocarcinoma	Moderate	Conventional	1	35	1	31	R_RF	NA	S-colon	MSI-L/S	0	No	Desert	k1
AMC-17CT-050	73	F	Adenocarcinoma	Moderate	Conventional	0	1176	0	31	R_RM	MSS	S-colon	MSI-L/S	0	Yes	Exhausted	k1
AMC-17CT-051	56	F	Adenocarcinoma	Moderate	Conventional	0	1176	0	997	R_B	MSI-High	A-colon	MSI-H	0	No	Exhausted	k2
AMC-17CT-052	62	M	Adenocarcinoma	Moderate	Conventional	0	1176	0	997	R_B	MSS	A-colon	MSI-L/S	0	Yes	Exhausted	k2
AMC-17CT-053	49	M	Adenocarcinoma	Moderate	Conventional	0	1175	1	202	R	MSS	Cecum	MSI-L/S	0	Yes	Desert	k3
AMC-17CT-054	61	F	Adenocarcinoma	Moderate	Conventional	0	1174	0	1021	R_RF	MSS	S-colon	MSI-L/S	0	Yes	Exhausted	k1
AMC-17CT-055	49	F	Adenocarcinoma	Moderate	Conventional	0	1173	0	979	R_B	MSS	S-colon	MSI-L/S	0	No	Desert	k3
AMC-17CT-056	75	M	Adenocarcinoma	Moderate	Conventional	1	748	1	354	R_B	MSS	Rectum	MSI-L/S	3	Yes	Exhausted	k1
AMC-17CT-057	56	M	Adenocarcinoma	Moderate	Conventional	0	1172	0	1166	R_B	MSS	S-colon	MSI-L/S	2	Yes	Exhausted	k1
AMC-17CT-058	47	F	Adenocarcinoma	Moderate	Conventional	0	1172	0	235	R_RF	MSS	Rectum	MSI-L/S	0	No	Exhausted	k1
AMC-17CT-059	61	M	Adenocarcinoma	Moderate	Conventional	0	1169	0	792	R_RF	MSS	Rectum	MSI-L/S	0	No	Exhausted	k1
AMC-17CT-060	84	M	Adenocarcinoma	Moderate	Conventional	0	1169	0	1163	R	MSS	Hepatic flexure	MSI-L/S	2.5	Yes	Active	k3
AMC-17CT-062	56	M	Adenocarcinoma	Moderate	Conventional	0	1168	1	206	R_B	MSI-Low	S-colon	MSI-L/S	0.5	No	Active	k2
AMC-17CT-063	51	M	Adenocarcinoma	Moderate	Conventional	1	937	1	616	R	MSS	Rectum	MSI-L/S	0.5	No	Active	k1
AMC-17CT-065	75	F	Adenocarcinoma	Poor	Conventional	0	1167	0	244	R	MSI-Low	S-colon	MSI-L/S	0	No	Exhausted	k1
AMC-17CT-066	47	M	Adenocarcinoma	Moderate	Conventional	0	1168	1	314	R	MSS	Rectum	MSI-L/S	2	Yes	Exhausted	k1
AMC-17CT-068	62	F	Adenocarcinoma	Moderate	Conventional	1	978	0	876	R_B	MSS	S-colon	MSI-L/S	0	No	Desert	k3
AMC-17CT-069	60	F	Adenocarcinoma	Moderate	Conventional	0	1165	0	958	R_RF	MSS	Rectum	MSI-L/S	2	Yes	Desert	k1
AMC-17CT-072	60	M	Adenocarcinoma	Moderate	Conventional	0	1162	0	16	R	MSS	S-colon	MSI-L/S	0	No	Desert	k3
AMC-17CT-073	53	M	Adenocarcinoma	Moderate	Conventional	1	915	1	31	R	MSS	Rectum	MSI-L/S	0	Yes	Exhausted	k1
AMC-17CT-074	55	M	Adenocarcinoma	Moderate	Conventional	0	1161	0	5	R	MSS	Cecum	MSI-L/S	0	Yes	Desert	k1
AMC-17CT-075	66	M	Adenocarcinoma	Moderate	Conventional	0	1158	1	19	R	MSS	Rectum	MSI-L/S	0.5	Yes	Desert	k4
AMC-17CT-078	59	M	Adenocarcinoma	Moderate	Conventional	0	1153	0	993	R	MSS	S-colon	MSI-L/S	0	No	Desert	k1
AMC-17CT-079	83	F	Adenocarcinoma	Moderate	Mucious and conventional	0	1153	0	923	R_RM	MSS	Rectum	MSI-L/S	0	Yes	Active	k4
AMC-17CT-080	60	F	Adenocarcinoma	Moderate	Conventional	0	1152	0	848	R_RF	MSS	S-colon	MSI-L/S	0	Yes	Desert	k1
AMC-17CT-081	56	F	Adenocarcinoma	Moderate	Conventional	0	1154	0	34	R	MSS	A-colon	MSI-L/S	0.5	Yes	Desert	k4
AMC-17CT-082	61	F	Adenocarcinoma	Well	Conventional	0	1148	0	953	R_RM	MSS	A-colon	MSI-L/S	0	Yes	Desert	k3
AMC-17CT-083	60	M	Adenocarcinoma	Moderate	Conventional	0	1148	0	556	R_RF	MSS	S-colon	MSI-L/S	0.5	No	Exhausted	k1
AMC-17CT-084	72	M	Adenocarcinoma	Moderate	Conventional	0	1146	0	498	R	MSS	Rectum	MSI-L/S	0	Yes	Exhausted	k2
AMC-17CT-085	50	M	Adenocarcinoma	Moderate	Conventional	0	1146	0	232	R	MSS	S-colon	MSI-L/S	0	No	Exhausted	k4
AMC-17CT-086	74	M	Adenocarcinoma	Moderate	Conventional	0	1141	0	463	R_B	MSS	Rectum	MSI-L/S	0	No	Desert	k1
AMC-17CT-088	88	M	Adenocarcinoma	Moderate	Conventional	0	1140	0	517	R_B	MSS	S-colon	MSI-L/S	0	Yes	Desert	k1
AMC-17CT-089	64	F	Adenocarcinoma	Moderate	Conventional	0	1139	0	1022	R	MSS	A-colon	MSI-L/S	2.5	Yes	Active	k2
AMC-17CT-090	50	F	Adenocarcinoma	Moderate	Conventional	0	1139	0	449	R	MSS	S-colon	MSI-L/S	0	Yes	Exhausted	k2
AMC-17CT-091	72	M	Adenocarcinoma	Moderate	Signet ring cell	1	445	0	351	RF	MSS	Rectum	MSI-L/S	0	No	Active	k2
AMC-17CT-093	82	F	Adenocarcinoma	Poor	Conventional	1	782	1	376	RF	MSS	A-colon	MSI-L/S	0	No	Active	k1
AMC-17CT-095	75	M	Adenocarcinoma	Moderate	Conventional	0	1132	0	978	R	MSS	S-colon	MSI-L/S	1	Yes	Desert	k2
AMC-17CT-096	56	F	Adenocarcinoma	Moderate	Conventional	0	1132	0	1002	R	MSS	D-colon	MSI-L/S	0	Yes	Active	k3
AMC-17CT-097	58	F	Adenocarcinoma	Moderate	Conventional	0	1130	0	668	R	MSS	S-colon	MSI-L/S	0.5	Yes	Desert	k1
AMC-17CT-099	62	F	Adenocarcinoma	Moderate	Conventional	0	1117	0	960	R	MSS	S-colon	MSI-L/S	3	Yes	Active	k3
AMC-17CT-101	60	M	Adenocarcinoma	Moderate	Conventional	0	1112	0	1064	R	MSS	Rectum	MSI-L/S	1.5	Yes	Exhausted	k3
AMC-17CT-103	69	M	Adenocarcinoma	Moderate	Conventional	0	1111	0	1030	R	MSI-High	Cecum	MSI-H	3	Yes	Exhausted	k4
AMC-17CT-106	57	M	Adenocarcinoma	Moderate	Conventional	0	1110	0	986	R	MSS	S-colon	MSI-L/S	0	No	Desert	k1
AMC-17CT-107	57	M	Adenocarcinoma	Moderate	Conventional	0	1110	0	973	RF_RM	MSS	Rectum	MSI-L/S	2.5	Yes	Exhausted	k4
AMC-17CT-108	94	M	Adenocarcinoma	Moderate	Conventional	1	133	0	28	R_B	MSS	S-colon	MSI-L/S	0	Yes	Desert	k2
AMC-17CT-109	28	F	Adenocarcinoma	Moderate	Conventional	0	1109	1	937	RM_RF	MSS	S-colon	MSI-L/S	0	No	Desert	k4
AMC-17CT-111	67	M	Adenocarcinoma	Moderate	Conventional	0	1106	0	962	R_B	MSI-High	A-colon	MSI-H	2.5	Yes	Exhausted	k4
AMC-17CT-112	53	M	Adenocarcinoma	Moderate	Mucious and conventional	0	1105	0	932	R	MSS	T-colon	MSI-L/S	0	Yes	Desert	k4
AMC-17CT-113	53	M	Adenocarcinoma	Well	Conventional	0	1105	0	897	R	MSI-Low	S-colon	MSI-L/S	2	No	Desert	k1
AMC-17CT-114	48	M	Adenocarcinoma	Moderate	Conventional	0	1104	0	938	R_RF_RM	MSS	S-colon	MSI-L/S	0	Yes	Desert	k3
AMC-17CT-115	56	M	Adenocarcinoma	Moderate	Conventional	0	1104	0	1055	R_RF_RM	MSS	S-colon	MSI-L/S	0.5	Yes	Desert	k2
AMC-17CT-116	63	F	Adenocarcinoma	Moderate	Mucious and conventional	0	1103	0	991	R_RM	MSS	S-colon	MSI-L/S	0	Yes	Desert	k2
AMC-17CT-117	59	M	Adenocarcinoma	Well	Conventional	0	1103	0	1033	RF	MSS	S-colon	MSI-L/S	0	Yes	Desert	k2
AMC-17CT-120	69	F	Adenocarcinoma	Moderate	Conventional	0	1097	0	411	R_RM	MSS	A-colon	MSI-L/S	2	Yes	Active	k4
AMC-17CT-121	57	M	Adenocarcinoma	Moderate	Conventional	0	1096	0	973	RF	MSS	S-colon	MSI-L/S	0	Yes	Active	k1
AMC-17CT-123	48	F	Adenocarcinoma	Moderate	Conventional	1	170	1	16	R	MSS	Splenic flexure	MSI-L/S	0.5	Yes	Desert	k1
AMC-17CT-125	75	M	Adenocarcinoma	Moderate	Conventional	1	521	1	22	R	MSS	A-colon	MSI-L/S	0	No	Exhausted	k1
AMC-17CT-131	68	M	Adenocarcinoma	Moderate	Mucious and conventional	1	661	1	183	R	MSS	A-colon	MSI-L/S	0	No	Exhausted	k4
AMC-17CT-135	74	M	Adenocarcinoma	Moderate	Conventional	0	1081	0	886	R_B	MSS	Rectum	MSI-L/S	0.5	Yes	Active	k2
AMC-17CT-138	36	M	Adenocarcinoma	Moderate	Conventional	0	1078	0	962	R	MSS	Rectum	MSI-L/S	0	Yes	Exhausted	k4
AMC-17CT-139	73	F	Adenocarcinoma	Moderate	Conventional	0	1077	0	828	R	MSS	A-colon	MSI-L/S	0	Yes	Active	k4
AMC-17CT-146	61	M	Adenocarcinoma	Moderate	Conventional	1	538	0	463	R_B	MSS	Rectum	MSI-L/S	0	No	Desert	k3
AMC-17CT-147	48	F	Adenocarcinoma	Well	Conventional	0	1071	0	787	R	MSI-High	T-colon	MSI-H	0	No	Exhausted	k4
