# Supplementary figures and images for: Immuno-genomic classification of colorectal cancer organoids reveals cancer cells with intrinsic immunogenic properties associated with patient survival
Source: J Exp Clin Cancer Res. 2021 Jul 13;40:230. doi: 10.1186/s13046-021-02034-1 (PMC8276416; doi:10.1186/s13046-021-02034-1)

Fig. S1

a

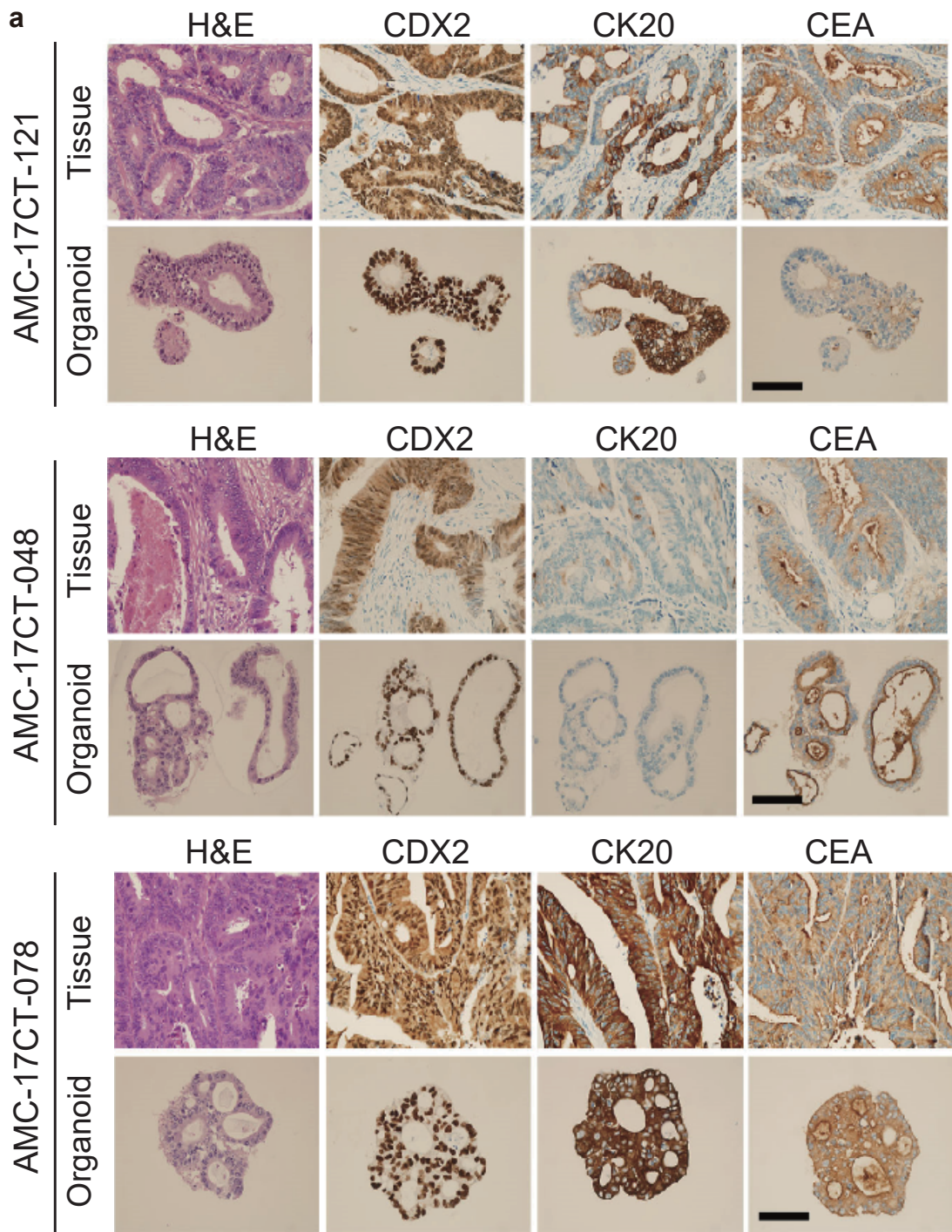

b

Variants detected from colorectal cancer organoids

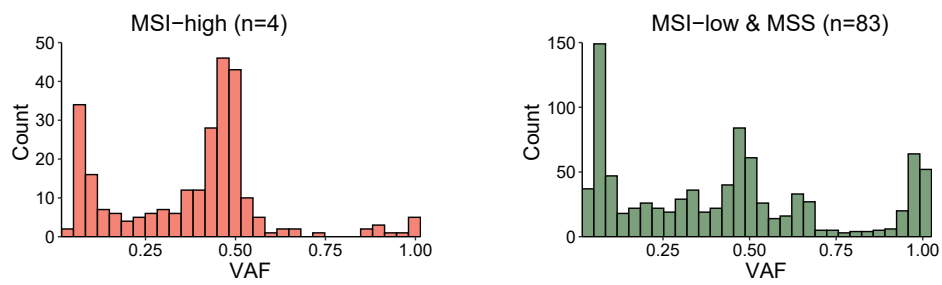

Supplement: Supplementary file 2 — Additional file 2 Supplementary Fig. 1. (A) Histology and immunohistochemistry of the established CCOs and the corresponding primary tumors. (B) Distribution of variant allele fraction (VAF) of detected variants in the MSI-high group and the MSI-low/MSS group. CCO, colorectal cancer organoid; MSI, microsatellite instability; MSS, microsatellite stable; H&E, hematoxylin and eosin. [file 13046_2021_2034_MOESM2_ESM.pdf]

Fig. S2

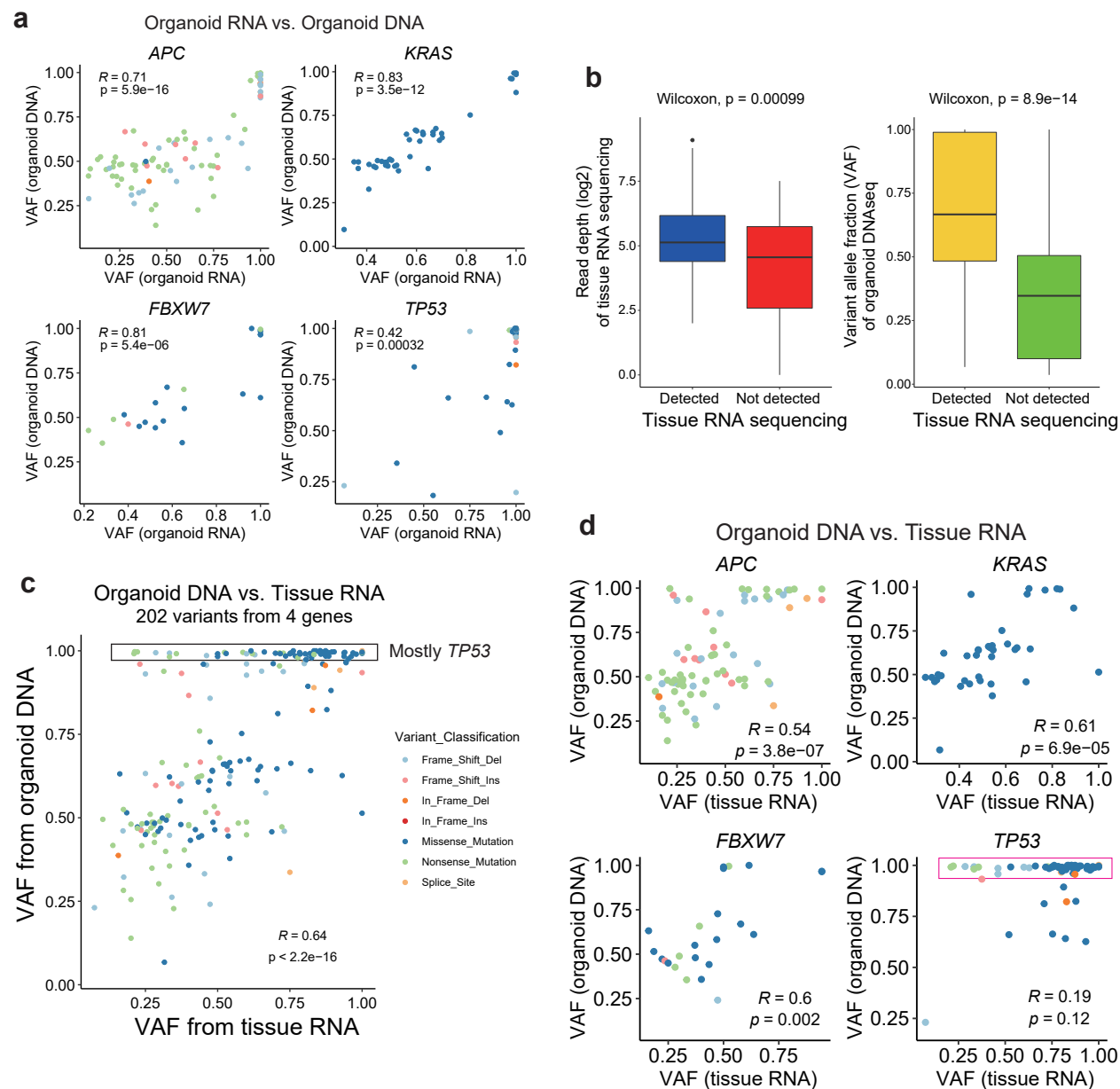

Supplement: Supplementary file 3 — Additional file 3 Supplementary Fig. 2. (A) Spearman’s correlation of variant allele fractions (VAFs) between organoid RNA and organoid DNA. (B) Characteristics of variants detected in organoid DNA and not in tissue RNA; the variants detected in organoids only were associated with low read depth in primary tissue sequences or subclonal events based on organoid sequencing (Wilcoxon rank-sum test). (C) Spearman’s correlation of VAFs between organoid DNA and tissue RNA. (D) Spearman’s correlation of VAF in each gene between organoid DNA and tissue RNA. [file 13046_2021_2034_MOESM3_ESM.pdf]

Fig. S4

a

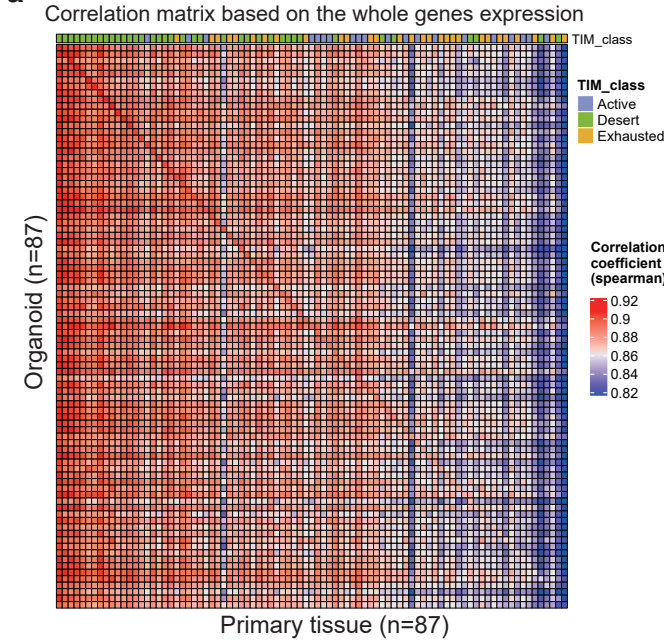

b

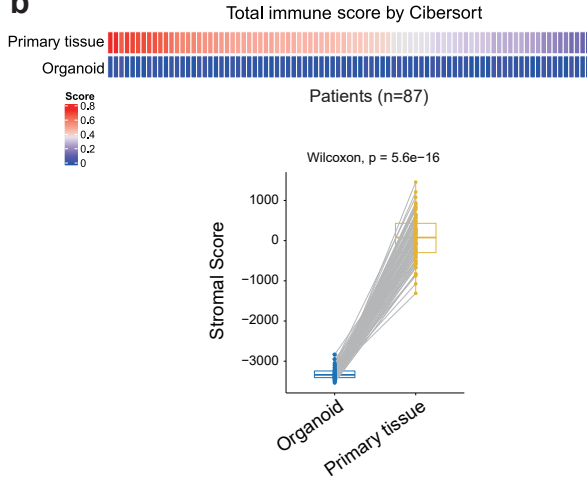

c

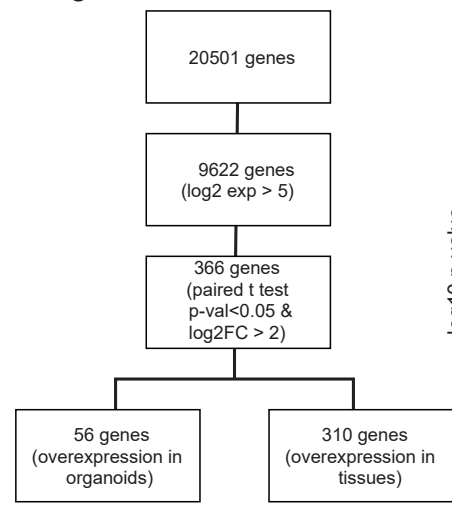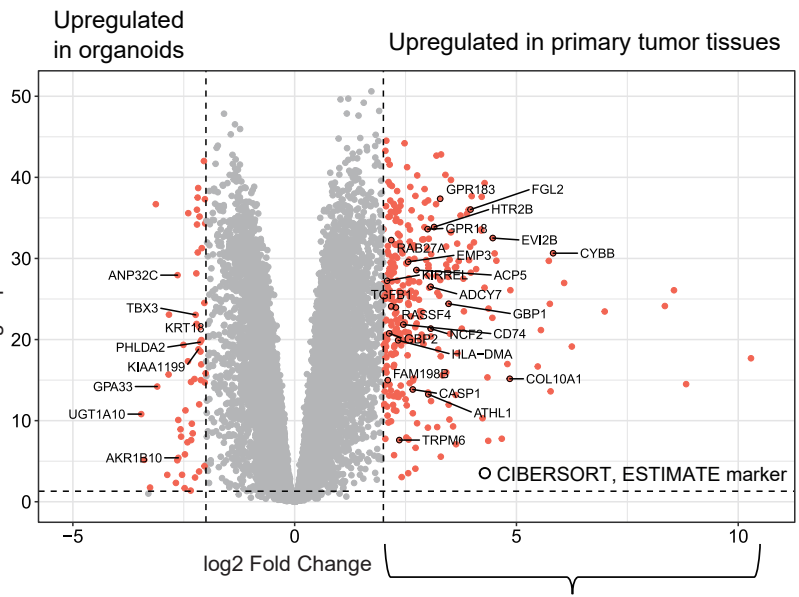

d

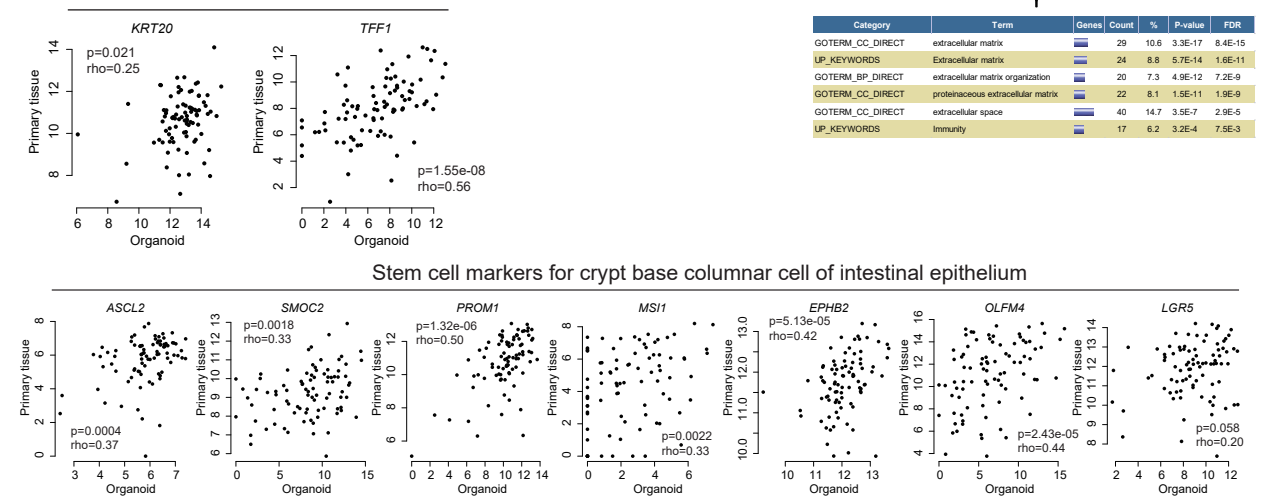

Supplement: Supplementary file 5 — Additional file 5 Supplementary Fig. 4. (A) Spearman’s correlation coefficient matrix of CCOs versus primary tumor tissues. (B) Total immune score and stromal score of CCOs versus primary tumor tissues. (C) Genes that are differentially expressed between CCOs and primary tumor tissues. (D) Spearman correlation of the expression of differentiation or stem cell markers in CCOs versus primary tumor tissues. CCO, colorectal cancer organoid; TIM, tumor immune microenvironment. [file 13046_2021_2034_MOESM5_ESM.pdf]

Fig. S6

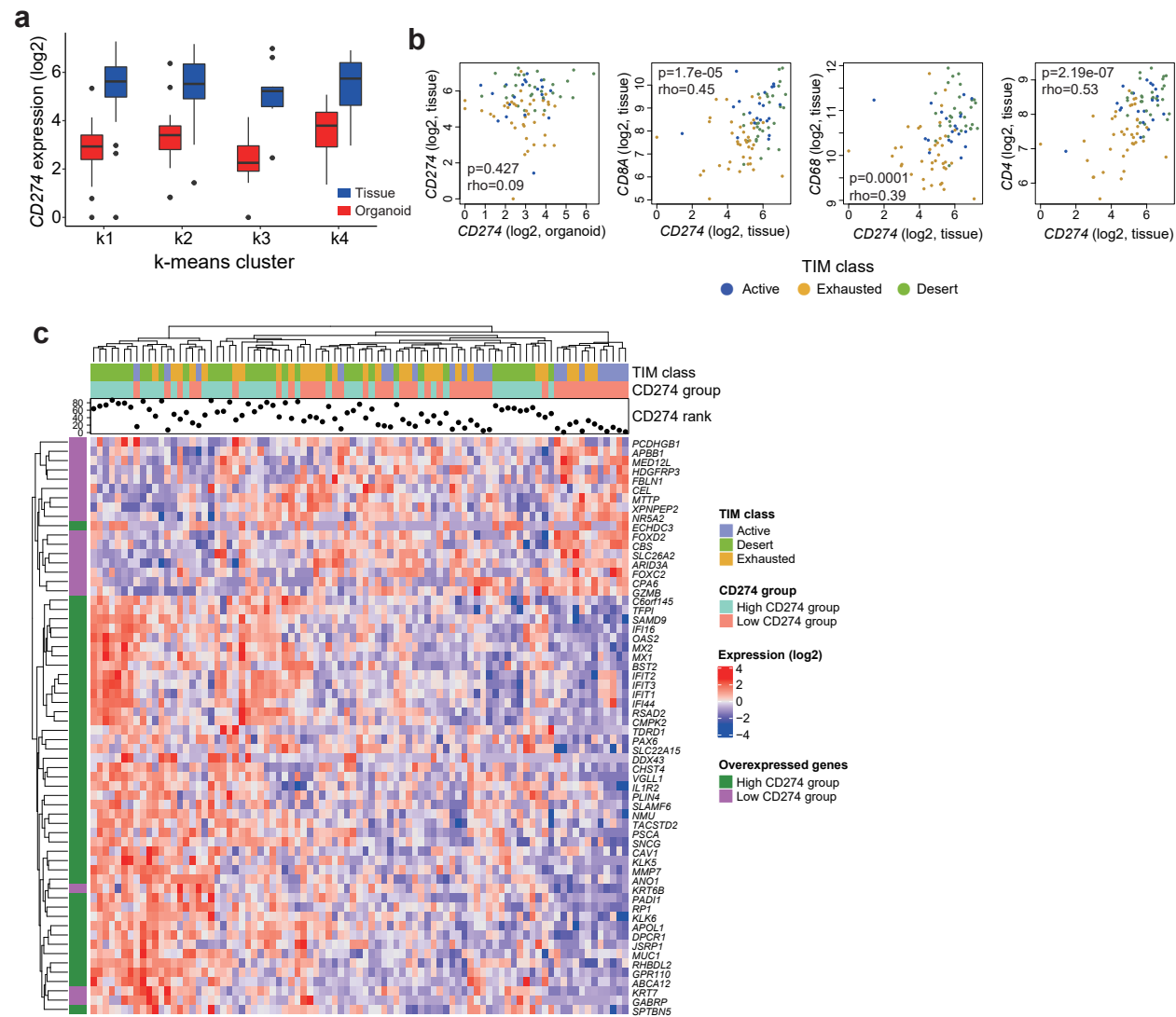

Supplement: Supplementary file 7 — Additional file 7 Supplementary Fig. 6. (A) PDL1 (CD274) expression levels in CCOs and primary tumor tissues. (B) PDL1 expression in primary tumor tissue was correlated with the expression of immune cell markers such as CD8A, CD68, and CD4, but was not correlated with PDL1 expression in CCOs (Spearman correlation test). (C) Differentially expressed genes in CCOs based on cancer-intrinsic (organoid) PDL1 expression level. CCO, colorectal cancer organoid; TIM, tumor immune microenvironment. [file 13046_2021_2034_MOESM7_ESM.pdf]

Fig. S7

**a**

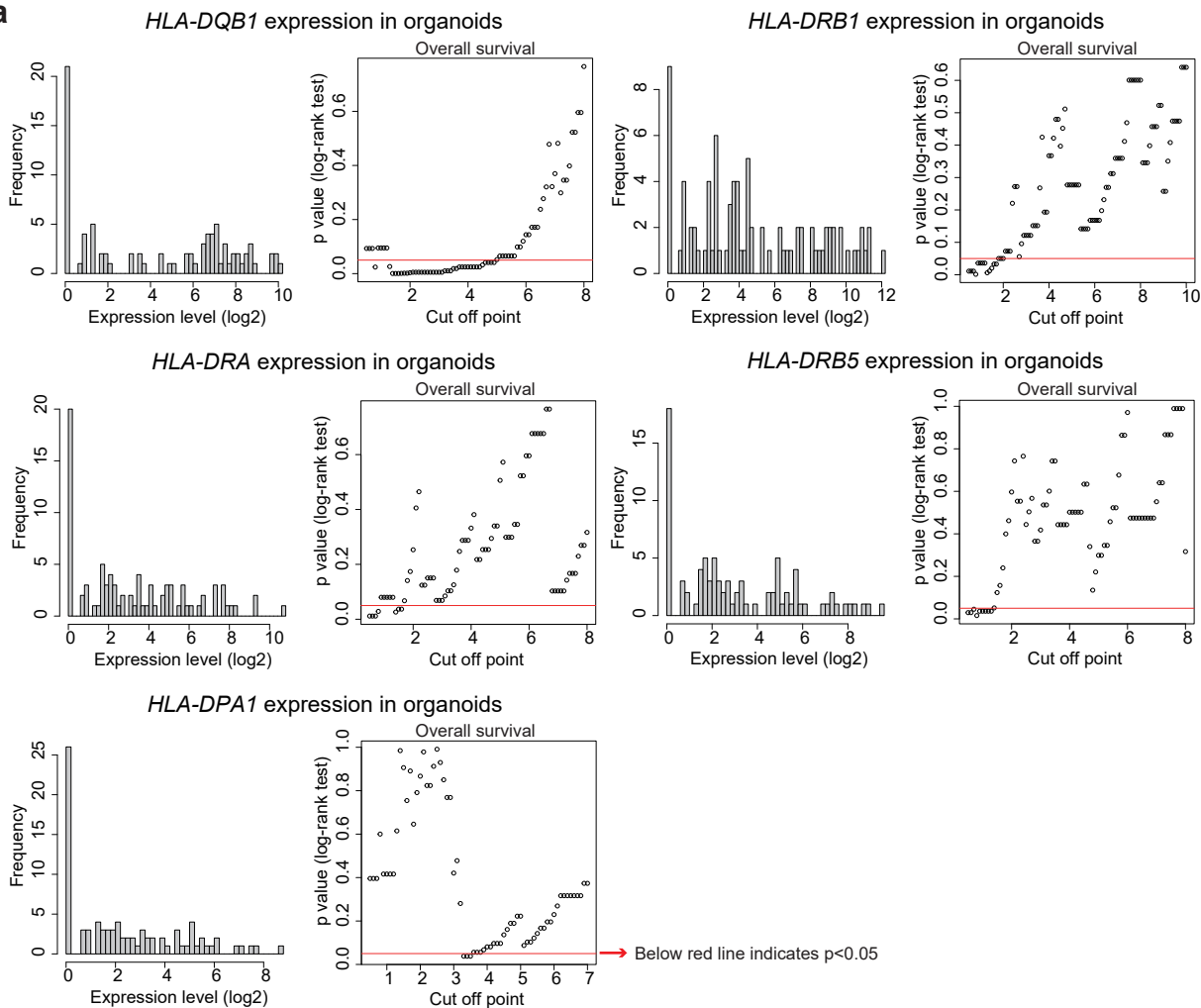

**b**

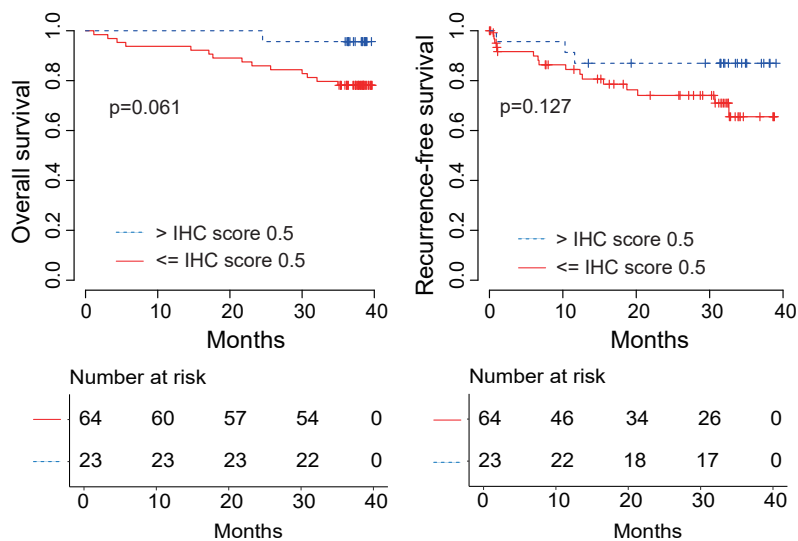

Supplement: Supplementary file 8 — Additional file 8 Supplementary Fig. 7. (A) Prognostic impact of gene expression in HLA class II in CCOs. (B) Patient survival based on HLA class II expression level using immunohistochemistry (IHC) in cancer cells from primary tissue (log-rank test). [file 13046_2021_2034_MOESM8_ESM.pdf]

Fig. S8

a

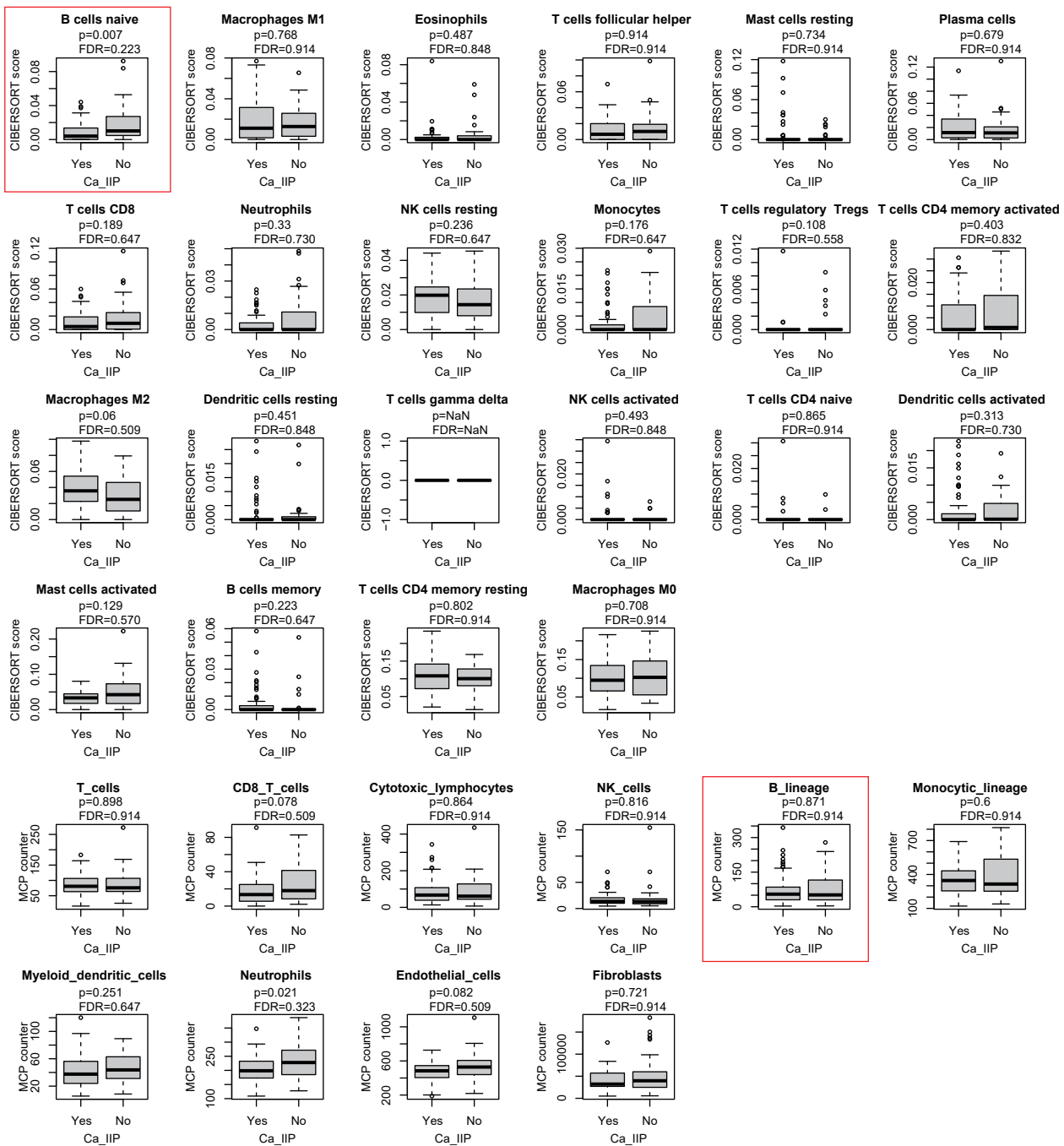

b

Ca-IIP group

No

Yes

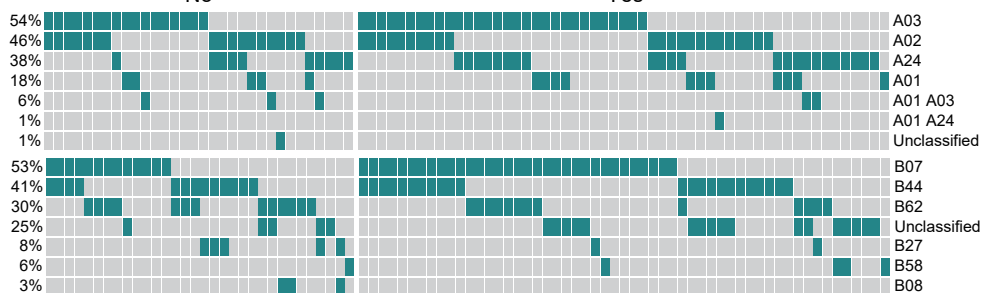

Supplement: Supplementary file 9 — Additional file 9 Supplementary Fig. 8. (A) Immune cell profiles of CIBERSORT and MCP based on the Ca-IIP group (Wilcoxon rank-sum test). B cells naive (CIBERSORT) score were lower in the Ca-IIP group. However, B lineage based on MCP score was not significant. (B) HLA class I supertype frequency based on CA-IIP group. Ca-IIP, cancer cells with intrinsic immunogenic properties. [file 13046_2021_2034_MOESM9_ESM.pdf]

Fig. S9

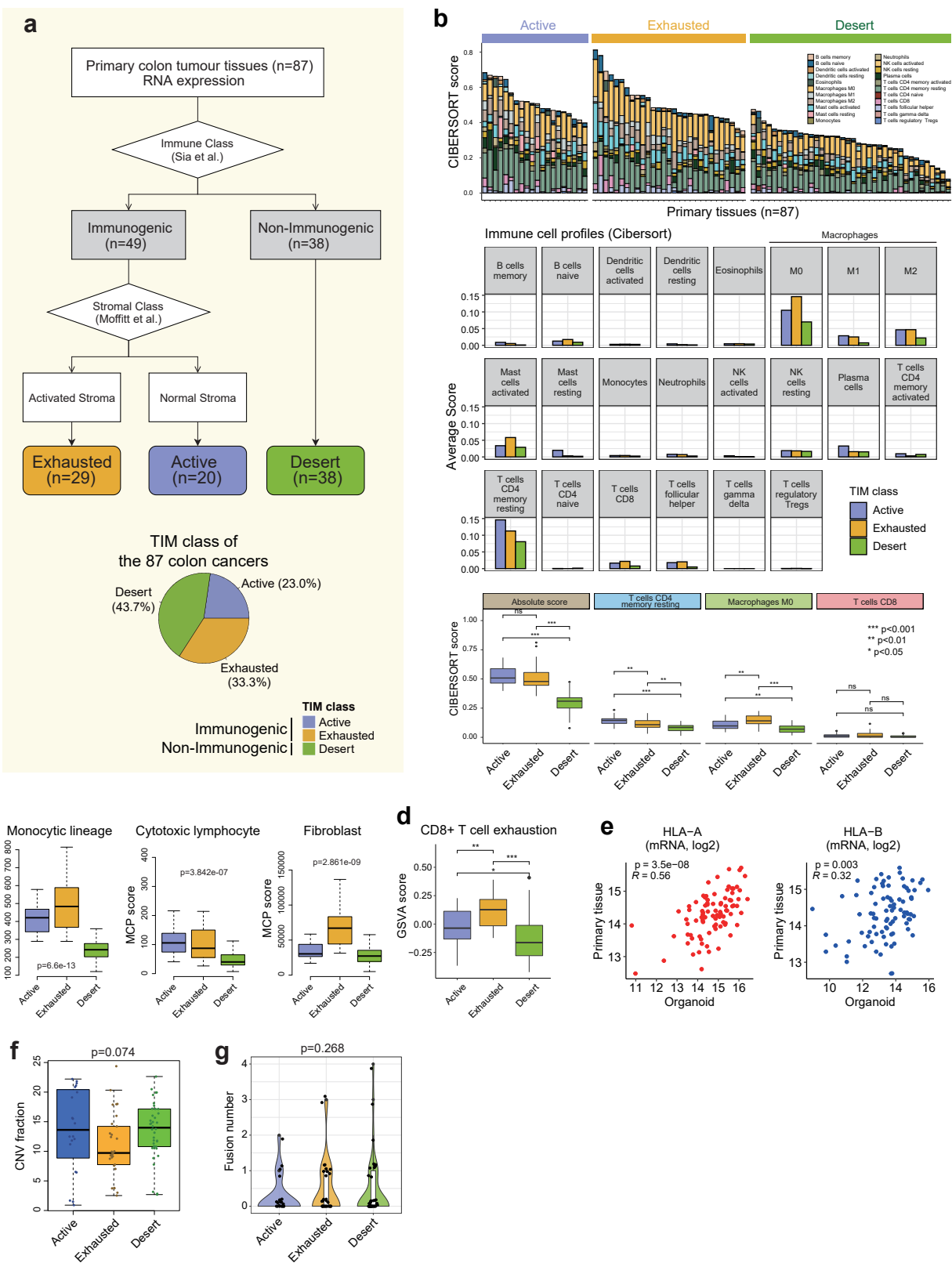

Supplement: Supplementary file 10 — Additional file 10 Supplementary Fig. 9. (A) Classification of the TIM using primary cancer tissue. (B) Immune cell types from CIBERSORT based on the TIM class (Wilcoxon rank-sum test). (C) Immune cell types using MCP score based on the TIM class (Kruskal Wallis test). (D) Validation of the exhausted TIM class based on CD8+ T cell exhaustion score using GSVA analysis (Wilcoxon rank-sum test). (E) Significant correlation of HLA-I expression in CCOs versus primary tumor tissues (Spearman correlation test). (F) Fraction of copy number variation (CNV) based on the TIM class (Kruskal Wallis test). (G) Fusion number based on the TIM class (Kruskal Wallis test). TIM, tumor immune microenvironment; CCO, colorectal cancer organoid. [file 13046_2021_2034_MOESM10_ESM.pdf]

Fig. S10

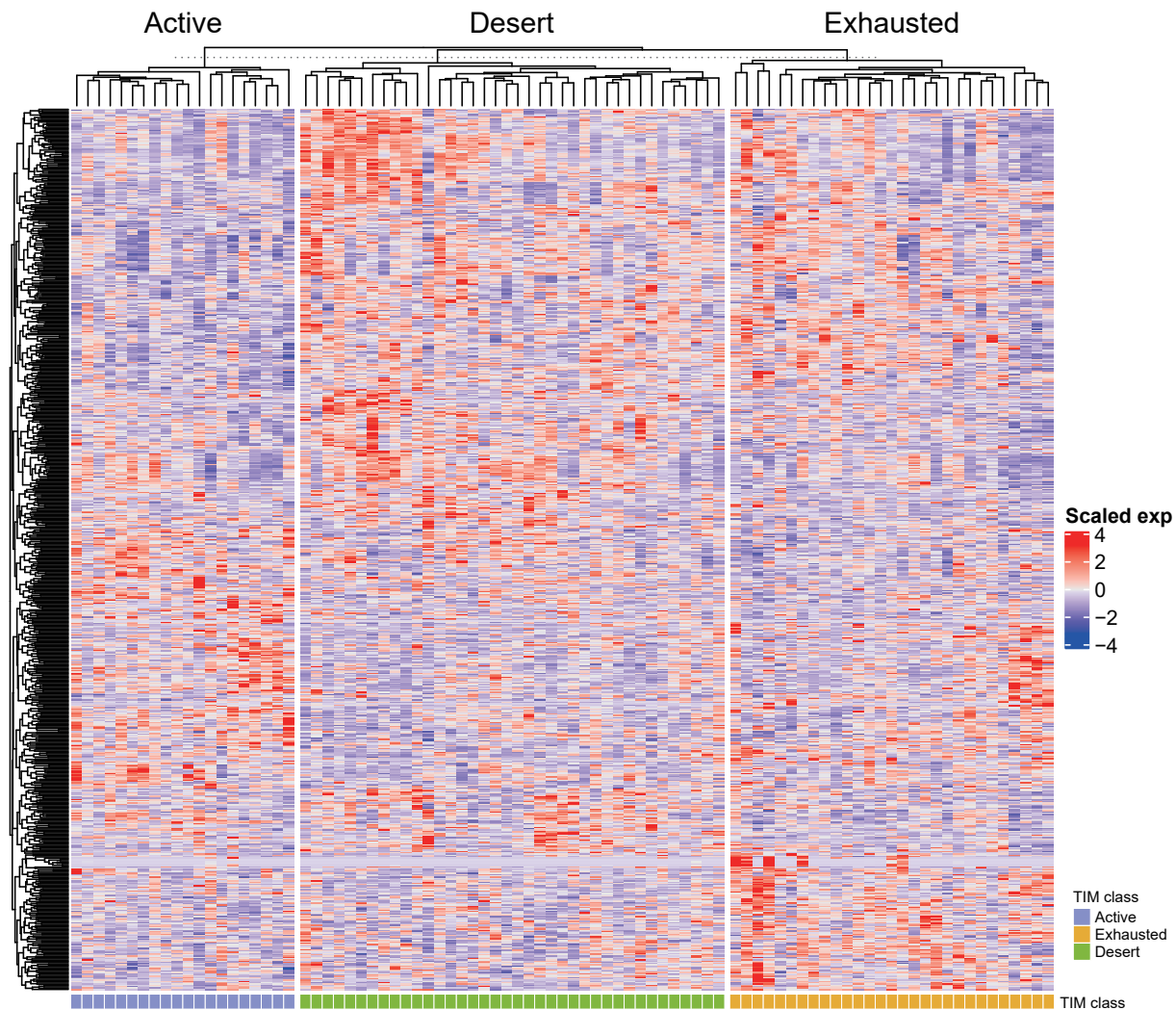

Supplement: Supplementary file 11 — Additional file 11 Supplementary Fig. 10. No significant differential gene expression in organoids based on the tumor immune microenvironment (TIM) class of primary tissues (FDR q > 0.25 by one-way ANOVA test). [file 13046_2021_2034_MOESM11_ESM.pdf]

Fig. S12

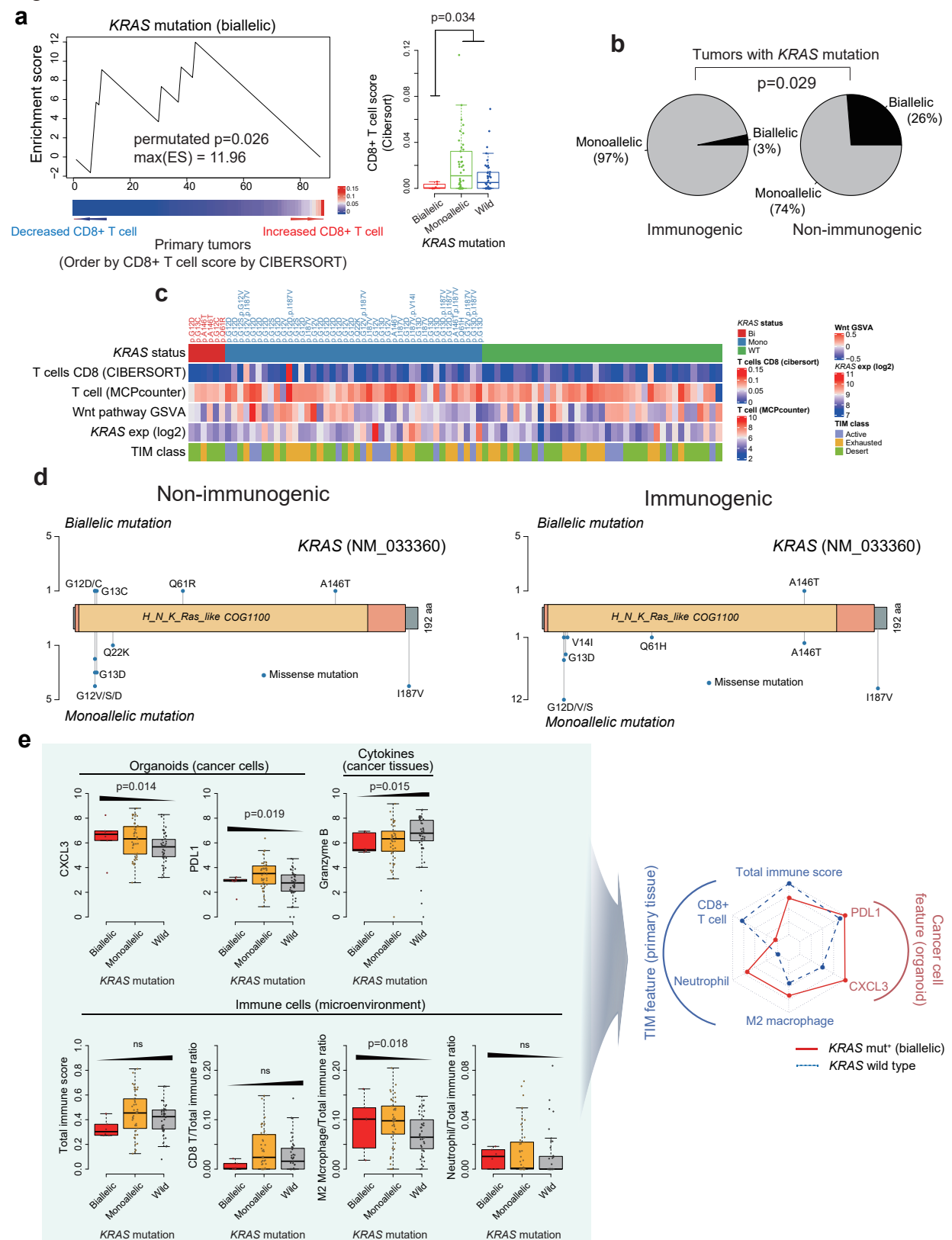

Supplement: Supplementary file 13 — Additional file 13 Supplementary Fig. 12. (A) Enrichment of KRAS (biallelic) mutations in tumors with decreased CD8+ T cells (10,000 randomly permutated p values and one-sided Wilcoxon rank sum test). (B) Increased frequency of KRAS biallelic mutation in non-immunogenic (immuno-desert TIM) tumors (Fisher’s exact test). (C, D) KRAS mutation status and TIM. (E) Characteristics of gene expression and infiltrating immune cell types based on the KRAS mutation status (Spearman’s correlation test). TIM, tumor immune microenvironment. [file 13046_2021_2034_MOESM13_ESM.pdf]

Fig. S13

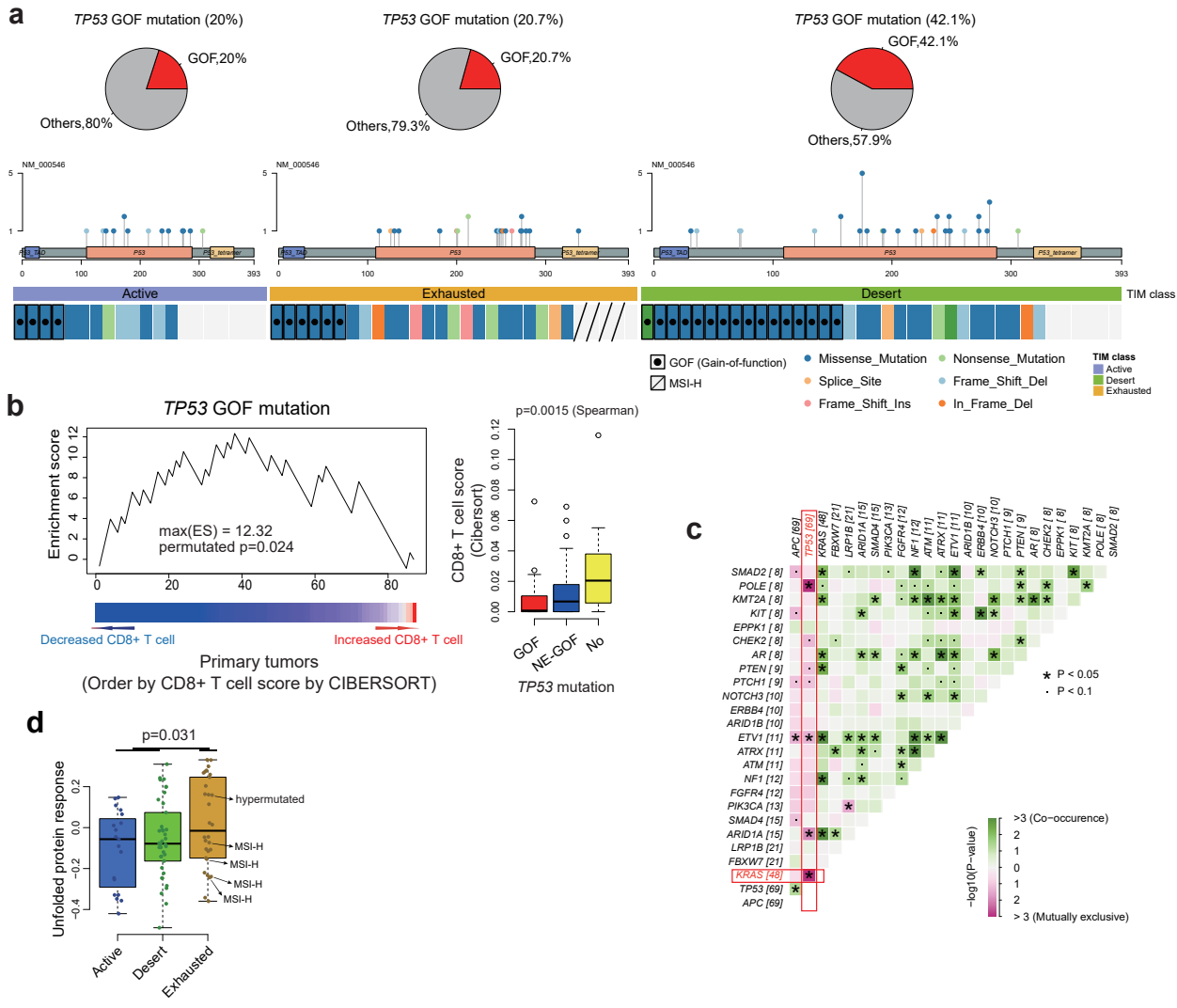

Supplement: Supplementary file 14 — Additional file 14 Supplementary Fig. 13. (A) Frequency of TP53 gain-of-function (GOF) mutation based on the tumor immune microenvironment (TIM) class, showing a high frequency of TP53 GOF mutations in the Desert group (p = 0.033, Fisher’s exact test). (B) Association between TP53 GOF mutations and decreased CD8+ T cell (10,000 random permutation test and Spearman’s correlation test). (C) Tendency for the exclusive occurrence of TP53 and KRAS mutation. (D) Enriched unfolded protein response pathway (GSVA score) in the immune-exhausted group (Wilcoxon rank-sum test). [file 13046_2021_2034_MOESM14_ESM.pdf]

Fig. S14

**a**

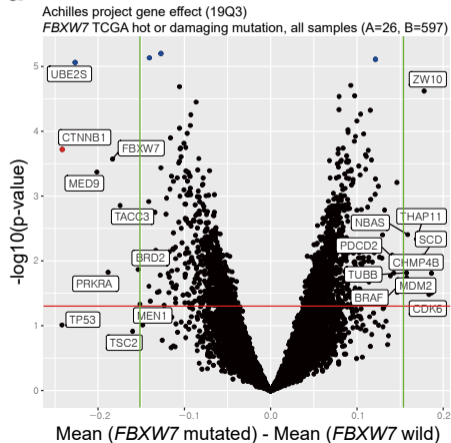

**b**

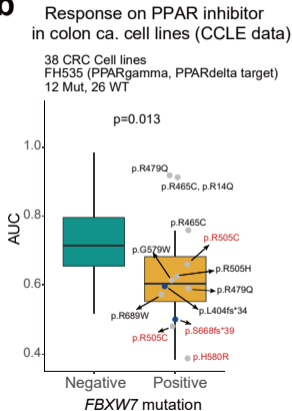

**c**

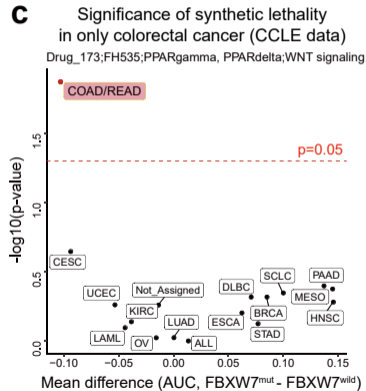

Supplement: Supplementary file 15 — Additional file 15 Supplementary Fig. 14. (A) Screening of synthetic lethality target of FBXW7 mutation using the DepMap dataset. (B, C) Sensitivity of the FH535 molecule in colorectal cancer cell lines with FBXW7 mutation (Wilcoxon rank sum test). [file 13046_2021_2034_MOESM15_ESM.pdf]

Fig. S15

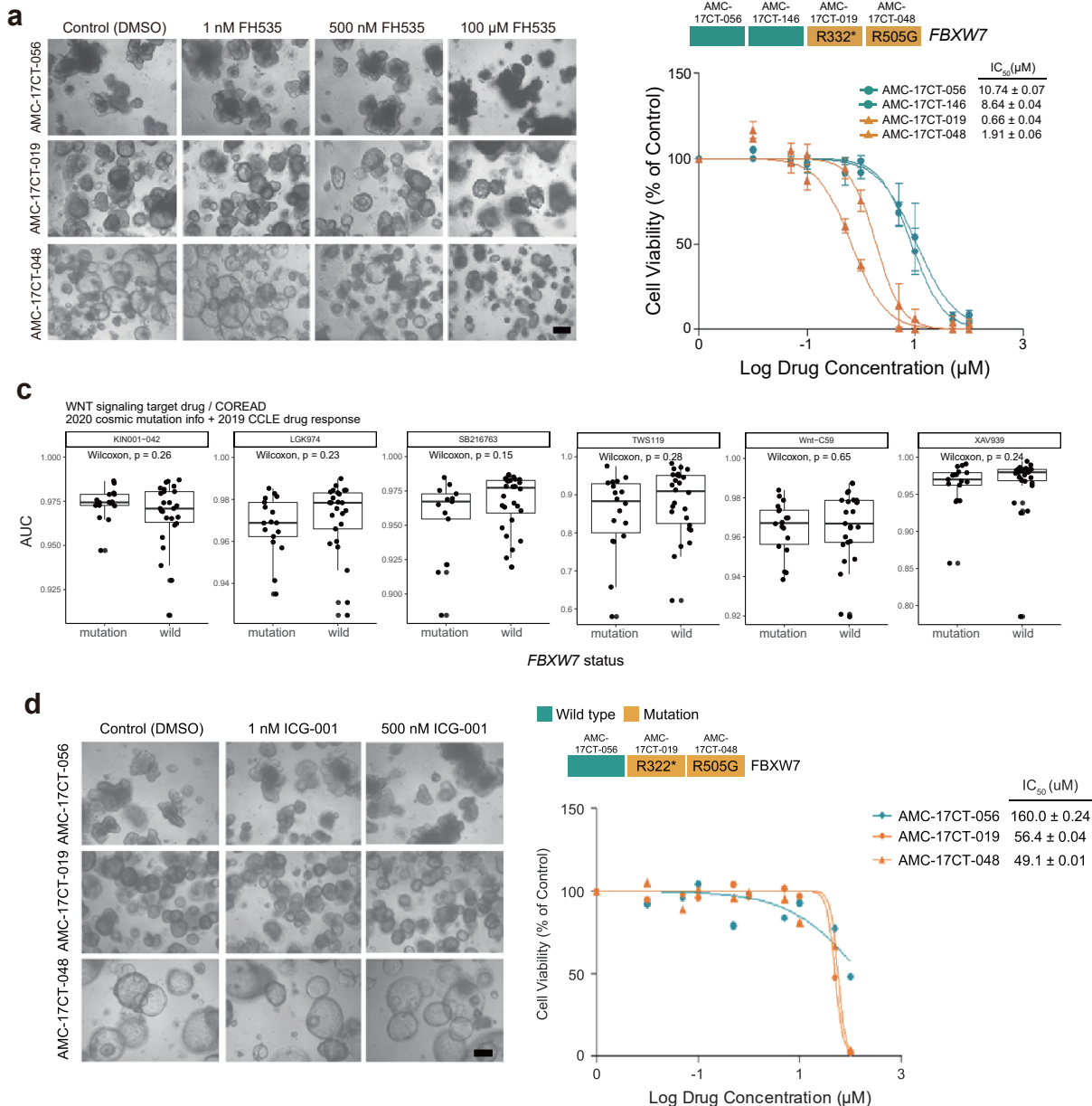

Supplement: Supplementary file 16 — Additional file 16 Supplementary Fig. 15. (A) FH535 induced cell death and destroyed the organoid structure in the two CCOs with FBXW7 mutation (AMC-17CT-019 and AMC-17CT-048). (B) Sensitivity of the FH535 molecule in CCOs with FBXW7 mutation (Wilcoxon-rank sum test). (C) Sensitivities of various WNT signaling drugs targets showing non-significant responses to drugs targeting WNT pathways in colorectal cancer cells with FBXW7 mutation. (D) CCOs with FBXW7 mutation also showed non-significant responses to ICG-001, which also targets the WNT/β-catenin pathway, suggesting that FH535 has a different mechanism action in colorectal cancers harboring the FBXW7 mutation. CCO, colorectal cancer organoid. [file 13046_2021_2034_MOESM16_ESM.pdf]
