# Supplementary material for: Immuno-genomic classification of colorectal cancer organoids reveals cancer cells with intrinsic immunogenic properties associated with patient survival
Source: J Exp Clin Cancer Res. 2021 Jul 13;40:230. doi: 10.1186/s13046-021-02034-1 (PMC8276416; doi:10.1186/s13046-021-02034-1)

Fig. S3

a

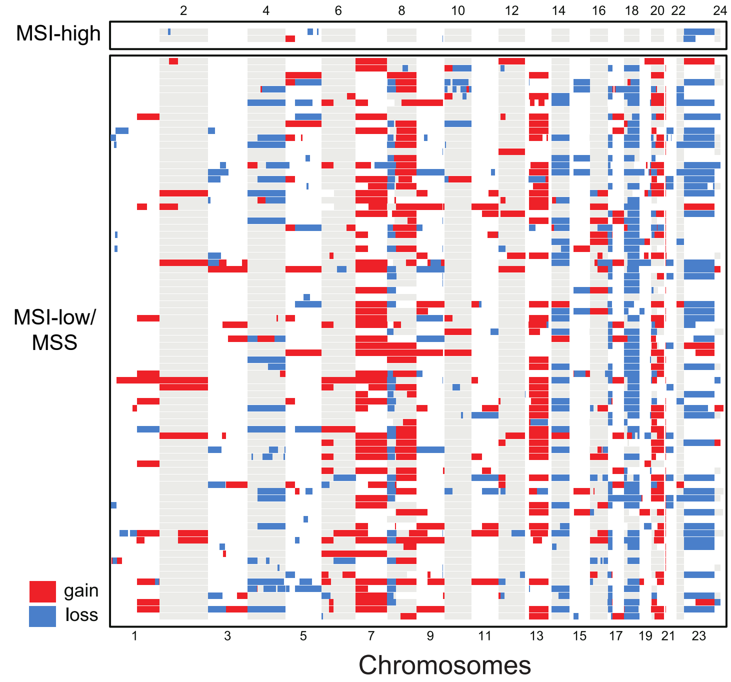

b

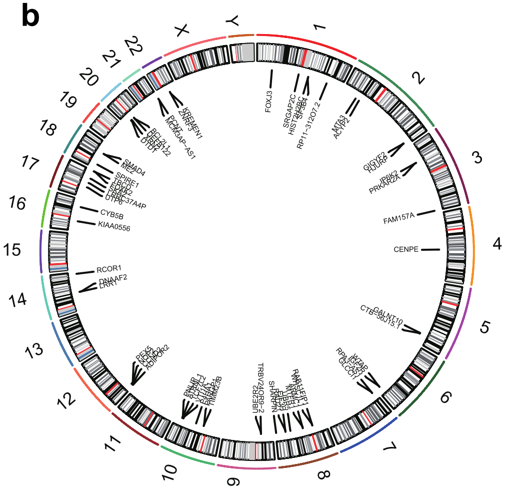

PEX5--NOP2	FOXJ3--EPB41L1
IP6K2--PRKAR2A	HJURP--GIGYF2
KIF3B--COMMD7	VTI1A--PNLIP
XRN2--RP11-560A15.3	RPA3-AS1--GLCCI1
FAM157A--SPIRE1	TM9SF4--DTD1
WHSC1L1--DNAAF2	TPX2--DEFB122
RAD21--EIF3H	TPX2--HCK
ZNRF3--KREMEN1	MCM3AP-AS1--PCNT
ME2--SMAD4	UBE2R2--TRBV24OR9-2
ADIPOR2--CCND2	CYB5B--KIAA0556
TCF7L2--ATRNL1	MTA3--ACYP2
CENPE--SKA2	LRRC37A4P--P4HA1
SHARPIN--UBR5	TIMM23B--PARGP1
TOP1--ARFGEF2	HIST2H2BC--SF3B4
BCL2L1--EFCAB8	RP11-312O7.2--SRGAP2C
RAB11FIP1--UTP6	RCOR1--LRR1
GALNT10--CTB-56J15.1	
ARMC1--MTFR1	
FOXK2--TBCD	
IGF2R--WTAP	

c

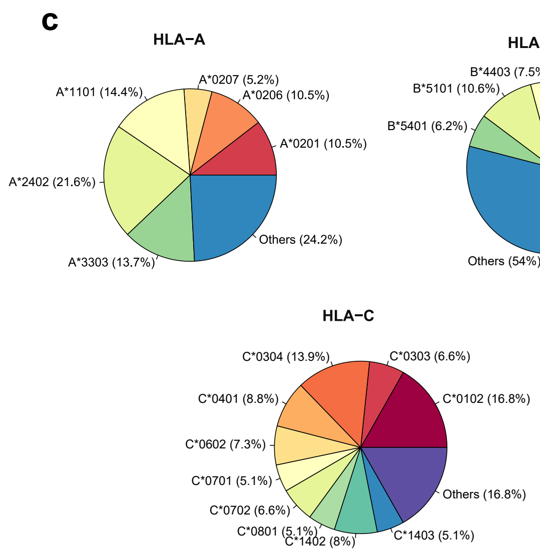

d

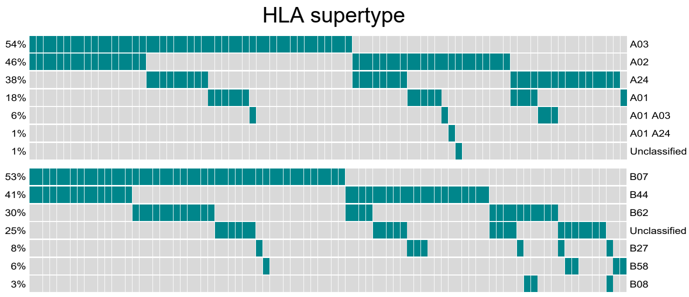

Supplement: Supplementary file 4 — Additional file 4 Supplementary Fig. 3. (A) Profiles of copy number variations with microsatellite instability (MSI) information in the 87 CCOs. (B) Circos plot of the detected fusions in the 87 CCOs. (C) HLA class I molecular typing and its frequency in 87 CCOs. (D) Frequency of the HLA supertype in 87 CCOs. CCO, colorectal cancer organoid. [file 13046_2021_2034_MOESM4_ESM.pdf]
