# Supplementary material for: Immuno-genomic classification of colorectal cancer organoids reveals cancer cells with intrinsic immunogenic properties associated with patient survival
Source: J Exp Clin Cancer Res. 2021 Jul 13;40:230. doi: 10.1186/s13046-021-02034-1 (PMC8276416; doi:10.1186/s13046-021-02034-1)
Supplement: Supplementary file 6 — Additional file 6 Supplementary Fig. 5. (A) Ki-67 immunohistochemistry in the primary tumor tissues and its interpretation by a pathologist and by image analysis. (B) Significant correlation of the Ki-67 proliferation index between the pathologist’s result and image analysis. (C) No correlations in Ki-67 mRNA expression between bulk primary tissues including tumor microenvironment and cancer cells and CCOs. (D) Comparison of our molecular subgrouping and CMS classification in CCOs (Chi-squared test). CCO, colorectal cancer organoid. [file 13046_2021_2034_MOESM6_ESM.pdf]

Fig. S5

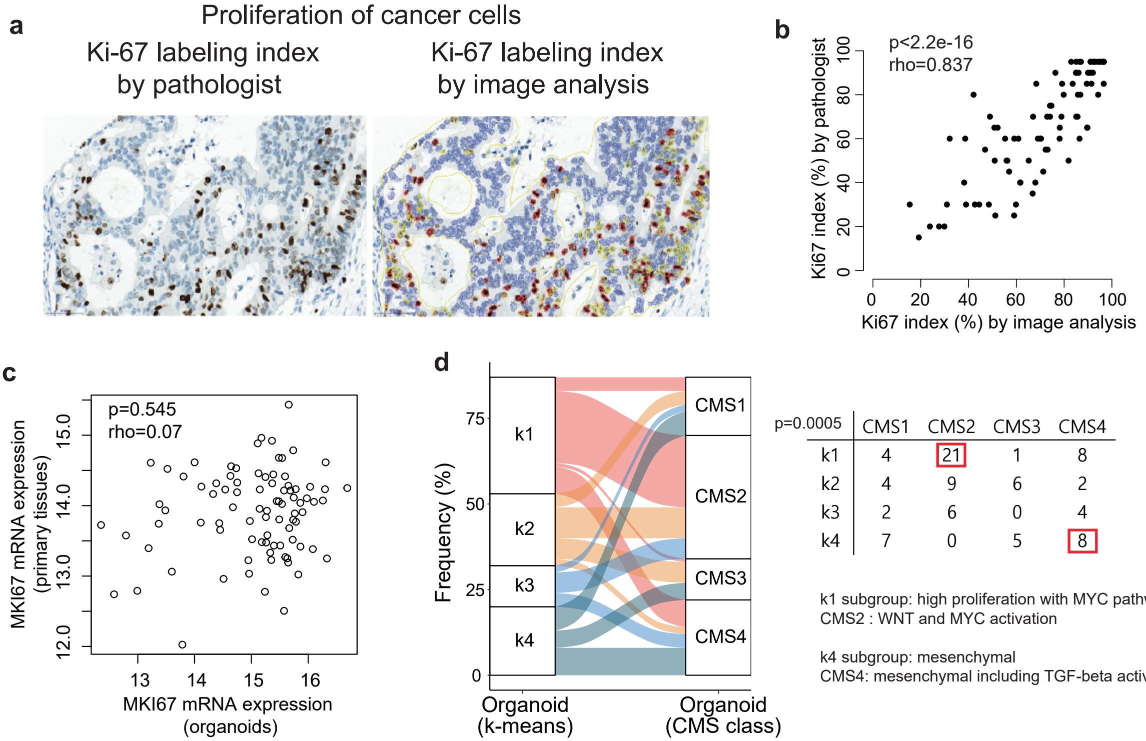

p=0.0005	CMS1	CMS2	CMS3	CMS4
k1	4	21	1	8
k2	4	9	6	2
k3	2	6	0	4
k4	7	0	5	8

k1 subgroup: high proliferation with MYC pathway activation
CMS2 : WNT and MYC activation

k4 subgroup: mesenchymal
CMS4: mesenchymal including TGF-beta activation
