# Supplementary material for: Immuno-genomic classification of colorectal cancer organoids reveals cancer cells with intrinsic immunogenic properties associated with patient survival
Source: J Exp Clin Cancer Res. 2021 Jul 13;40:230. doi: 10.1186/s13046-021-02034-1 (PMC8276416; doi:10.1186/s13046-021-02034-1)
Supplement: Supplementary file 12 — Additional file 12 Supplementary Fig. 11. (A) Pathway analysis using tissue RNA sequence data based on the tumor immune microenvironment (TIM) class. (B, C) Significance of Wnt/β-catenin signaling pathway in the non-immunogenic group using GSVA analysis (Wilcoxon rank-sum test). (D) APC mutation status based on the TIM class. (E) Significance of biallelic APC mutation and TIM class (Fisher’s exact test). (F) Expression of target genes belonging to the Wnt/beta-catenin pathway (Wilcoxon rank-sum test). [file 13046_2021_2034_MOESM12_ESM.pdf]

Fig. S11

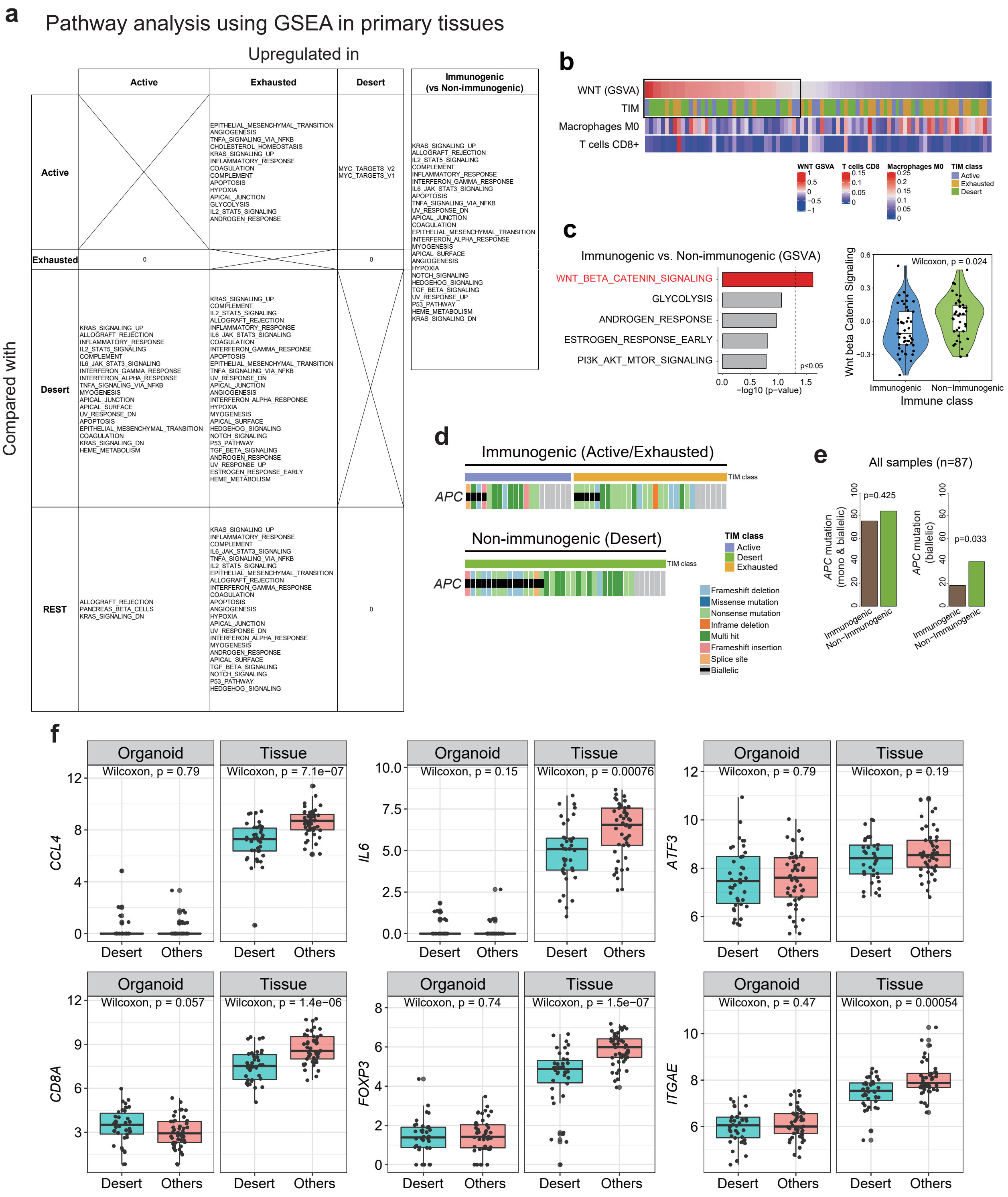

a
Pathway analysis using GSEA in primary tissues
Upregulated in
Active
Exhausted
Desert
Immunogenic (vs Non-immunogenic)
Compared with
Active
EPITHELIAL_MESENCHYMAL_TRANSITION
ANGIOGENESIS
TNFA_SIGNALING_VIA_NFKB
CHOLESTEROL_HOMEOSTASIS
KRAS_SIGNALING_UP
INFLAMMATORY_RESPONSE
COAGULATION
COMPLEMENT
APOPTOSIS
HYPOXIA
APICAL_JUNCTION
GLYCOLYSIS
IL2_STAT5_SIGNALING
ANDROGEN_RESPONSE
MYC_TARGETS_V2
MYC_TARGETS_V1
KRAS_SIGNALING_UP
ALLOGRAFT_REJECTION
IL2_STAT5_SIGNALING
COMPLEMENT
INFLAMMATORY_RESPONSE
INTERFERON_GAMMA_RESPONSE
IL6_JAK_STAT3_SIGNALING
APOPTOSIS
TNFA_SIGNALING_VIA_NFKB
UV_RESPONSE_DN
APICAL_JUNCTION
COAGULATION
EPITHELIAL_MESENCHYMAL_TRANSITION
INTERFERON_ALPHA_RESPONSE
MYOGENESIS
APICAL_SURFACE
ANGIOGENESIS
HYPOXIA
NOTCH_SIGNALING
HEDGEHOG_SIGNALING
TGF_BETA_SIGNALING
UV_RESPONSE_UP
P53_PATHWAY
HEME_METABOLISM
KRAS_SIGNALING_DN
Exhausted
0
0
Desert
KRAS_SIGNALING_UP
ALLOGRAFT_REJECTION
INFLAMMATORY_RESPONSE
IL2_STAT5_SIGNALING
COMPLEMENT
IL6_JAK_STAT3_SIGNALING
INTERFERON_GAMMA_RESPONSE
INTERFERON_ALPHA_RESPONSE
TNFA_SIGNALING_VIA_NFKB
MYOGENESIS
APICAL_JUNCTION
APICAL_SURFACE
UV_RESPONSE_DN
APOPTOSIS
EPITHELIAL_MESENCHYMAL_TRANSITION
COAGULATION
KRAS_SIGNALING_DN
HEME_METABOLISM
KRAS_SIGNALING_UP
COMPLEMENT
IL2_STAT5_SIGNALING
ALLOGRAFT_REJECTION
INFLAMMATORY_RESPONSE
IL6_JAK_STAT3_SIGNALING
COAGULATION
INTERFERON_GAMMA_RESPONSE
APOPTOSIS
EPITHELIAL_MESENCHYMAL_TRANSITION
TNFA_SIGNALING_VIA_NFKB
UV_RESPONSE_DN
APICAL_JUNCTION
ANGIOGENESIS
INTERFERON_ALPHA_RESPONSE
HYPOXIA
MYOGENESIS
APICAL_SURFACE
HEDGEHOG_SIGNALING
NOTCH_SIGNALING
P53_PATHWAY
TGF_BETA_SIGNALING
ANDROGEN_RESPONSE
UV_RESPONSE_UP
ESTROGEN_RESPONSE_EARLY
HEME_METABOLISM
REST
ALLOGRAFT_REJECTION
PANCREAS_BETA_CELLS
KRAS_SIGNALING_DN
KRAS_SIGNALING_UP
INFLAMMATORY_RESPONSE
COMPLEMENT
IL6_JAK_STAT3_SIGNALING
TNFA_SIGNALING_VIA_NFKB
IL2_STAT5_SIGNALING
EPITHELIAL_MESENCHYMAL_TRANSITION
ALLOGRAFT_REJECTION
INTERFERON_GAMMA_RESPONSE
COAGULATION
APOPTOSIS
ANGIOGENESIS
HYPOXIA
APICAL_JUNCTION
UV_RESPONSE_DN
INTERFERON_ALPHA_RESPONSE
MYOGENESIS
ANDROGEN_RESPONSE
APICAL_SURFACE
TGF_BETA_SIGNALING
NOTCH_SIGNALING
P53_PATHWAY
HEDGEHOG_SIGNALING
0
b
WNT (GSVA)
TIM
Macrophages M0
T cells CD8+
WNT GSVA
T cells CD8
Macrophages M0
TIM class
Active
Exhausted
Desert
c
Immunogenic vs. Non-immunogenic (GSVA)
WNT_BETA_CATENIN_SIGNALING
GLYCOLYSIS
ANDROGEN_RESPONSE
ESTROGEN_RESPONSE_EARLY
PI3K_AKT_MTOR_SIGNALING
p<0.05
−log10 (p−value)
Wilcoxon, p = 0.024
Wnt beta Catenin Signaling
Immunogenic
Non−Immunogenic
Immune class
d
Immunogenic (Active/Exhausted)
TIM class
APC
Non-immunogenic (Desert)
TIM class
APC
TIM class
Active
Desert
Exhausted
Frameshift deletion
Missense mutation
Nonsense mutation
Inframe deletion
Multi hit
Frameshift insertion
Splice site
Biallelic
e
All samples (n=87)
p=0.425
APC mutation (mono & biallelic)
p=0.033
APC mutation (biallelic)
Immunogenic
Non−Immunogenic
f
Organoid
Tissue
Wilcoxon, p = 0.79
Wilcoxon, p = 7.1e−07
CCL4
Wilcoxon, p = 0.15
Wilcoxon, p = 0.00076
IL6
Wilcoxon, p = 0.79
Wilcoxon, p = 0.19
ATF3
Wilcoxon, p = 0.057
Wilcoxon, p = 1.4e−06
CD8A
Wilcoxon, p = 0.74
Wilcoxon, p = 1.5e−07
FOXP3
Wilcoxon, p = 0.47
Wilcoxon, p = 0.00054
ITGAE
Desert
Others
